# Sebaceous immunobiology - skin homeostasis, pathophysiology, coordination of innate immunity and inflammatory response and disease associations

**DOI:** 10.3389/fimmu.2022.1029818

**Published:** 2022-11-10

**Authors:** Christos C. Zouboulis, Tom Coenye, Li He, Kenji Kabashima, Tetsuro Kobayashi, Catherin Niemann, Takashi Nomura, Attila Oláh, Mauro Picardo, Sven R. Quist, Hironobu Sasano, Marlon R. Schneider, Daniel Törőcsik, Sunny Y. Wong

**Affiliations:** ^1^ Departments of Dermatology, Venereology, Allergology and Immunology, Dessau Medical Center, Brandenburg Medical School Theodor Fontane and Faculty of Health Sciences Brandenburg, Dessau, Germany; ^2^ Laboratory of Pharmaceutical Microbiology, Ghent University, Ghent, Belgium; ^3^ Department of Dermatology, First Affiliated Hospital of Kunming Medical University, Kunming, Yunnan, China; ^4^ Department of Dermatology, Kyoto University Graduate School of Medicine, Kyoto, Japan; ^5^ Laboratory for Innate Immune Systems, RIKEN Center for Integrative Medical Sciences (IMS), Yokohama, Kanagawa, Japan; ^6^ Center for Molecular Medicine Cologne, CMMC Research Institute, University of Cologne, Cologne, Germany; ^7^ Center for Biochemistry, Medical Faculty, University of Cologne, Cologne, Germany; ^8^ Department of Physiology, Faculty of Medicine, University of Debrecen, Debrecen, Hungary; ^9^ San Gallicano Dermatologic Institute, IRCCS, Rome, Italy; ^10^ Department of Dermatology, Otto-von-Guericke University Magdeburg, Magdeburg, Germany; ^11^ Department of Pathology, Tohoku University School of Medicine, Sendai, Japan; ^12^ Institute of Veterinary Physiology, Faculty of Veterinary Medicine, University of Leipzig, Leipzig, Germany; ^13^ Department of Dermatology, Faculty of Medicine, University of Debrecen and ELKH-DE Allergology Research Group, Debrecen, Hungary; ^14^ Departments of Dermatology and Cell and Developmental Biology, University of Michigan, Ann Arbor, MI, United States

**Keywords:** sebaceous gland, sebocyte, hair follicle, developmental biology, stem cells, immunology, inflammation, sebaceous gland disease

## Abstract

This review presents several aspects of the innovative concept of sebaceous immunobiology, which summarizes the numerous activities of the sebaceous gland including its classical physiological and pathophysiological tasks, namely sebum production and the development of seborrhea and acne. Sebaceous lipids, which represent 90% of the skin surface lipids in adolescents and adults, are markedly involved in the skin barrier function and perifollicular and dermal innate immune processes, leading to inflammatory skin diseases. Innovative experimental techniques using stem cell and sebocyte models have clarified the roles of distinct stem cells in sebaceous gland physiology and sebocyte function control mechanisms. The sebaceous gland represents an integral part of the pilosebaceous unit and its status is connected to hair follicle morphogenesis. Interestingly, professional inflammatory cells contribute to sebocyte differentiation and homeostasis, whereas the regulation of sebaceous gland function by immune cells is antigen-independent. Inflammation is involved in the very earliest differentiation changes of the pilosebaceous unit in acne. Sebocytes behave as potent immune regulators, integrating into the innate immune responses of the skin. Expressing inflammatory mediators, sebocytes also contribute to the polarization of cutaneous T cells towards the Th17 phenotype. In addition, the immune response of the perifollicular infiltrate depends on factors produced by the sebaceous glands, mostly sebaceous lipids. Human sebocytes *in vitro* express functional pattern recognition receptors, which are likely to interact with bacteria in acne pathogenesis. Sex steroids, peroxisome proliferator-activated receptor ligands, neuropeptides, endocannabinoids and a selective apoptotic process contribute to a complex regulation of sebocyte-induced immunological reaction in numerous acquired and congenital skin diseases, including hair diseases and atopic dermatitis.

## 1 Introduction

Beyond the obvious interest in the sebaceous gland (SG) anatomy and function due to its major role in the pathogenesis of acne (1, 2), the most common inflammatory skin disorder (3), recent research has identified that the SG exhibits a pivotal role in several skin diseases and syndromes (4). At the turn of the century, the confirmation that human sebocytes, the cells that constitute the SG, control active sex hormone accumulation and metabolism in human skin (5) and produce neuropeptides, such as corticotropin-releasing hormone (CRH), which parallelly regulates the expression of androgen receptors (6), fueled the interest of the significance of the SG in the endocrine/neuroendocrine functions of the skin (7–13).

Moreover, supported by widely used human models (14, 15), evidence has lately been provided that sebocytes overtake the coordination of cellular communication inside the skin and are occasionally able to substitute professional inflammatory cells in the induction of innate immunity responses (2, 4). The - at least partial - elucidation of sebaceous hormone- and lipid-induced inflammation and its regulation (16–20) has widened our understanding for the involvement of the SG in skin inflammatory processes over the borders of acne, including psoriasis, atopic dermatitis, hidradenitis suppurativa and skin aging (2, 21–29), and has opened new signaling options for treatment of inflammatory skin and SG diseases (30, 31).

In addition to the revolution in our knowledge of SG-induced inflammatory signaling in the last 20 years, relevant developmental biology studies have identified an unexpected capacity of differentiated human sebocytes to overcome and reverse their lineage-specific differentiation upon certain signals (32). This exciting insight led to three different research directions; first towards the identification of sebocyte progenitor cells and their regulation (33–35), second the detection of aryl hydrocarbon receptor (AHR)-associated dysregulation of lineage differentiation of sebocyte progenitor cells into keratinocytes by endocrine disrupting environmental agents (36–39), and third the recognition of holocrine sebocyte secretion as a multistep, cell-specific lysosomal DNase2-mediated mode of programmed cell death associated with autophagy, which provides undifferentiated sebocytes precious nutritive materials and makes sebocytes self-sufficient (40–42). The latter is an invaluable characteristic of a skin gland, which is considered “the brain of the skin” by the authors of this review (15, 43, 44).

The present article reviews fundamental current knowledge on SG developmental biology and innate immunity and how SG contribute to skin homeostasis, pathophysiology, coordination of inflammatory skin response and disease prevention.

## 2 Developmental biology of the SG

### 2.1 SG stem and progenitor cells in development, homeostasis and pathologies

**Figure 1:**
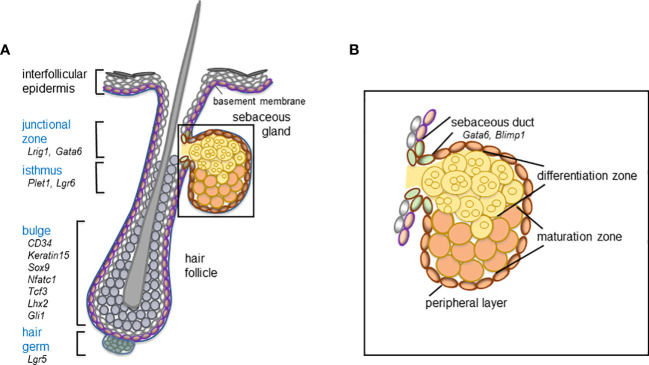
Schematic overview of the hair follicle stem and progenitor cell compartments (blue) **(A)** and distinct cell compartments comprising the mature sebaceous gland **(B) **including relevant marker molecules expressed by the distinct cell compartments of the mouse telogen hair follicle and the sebaceous duct.

The identification of skin epithelial stem cells and the isolation and functional characterization of multiple stem cell pools have led to a deeper understanding of their unique and complex role driving SG homeostasis and contributing to SG pathologies. During skin homeostasis, diverse stem cell pools are maintaining distinct epithelial compartments, but contribute to additional epithelial lineages following injuries (45). In particular, stem and progenitor cells localize to the bulge, hair germ, the isthmus and junctional zone of the hair follicle (46, 47) (**Figure 1**). Innovative techniques, including *in vivo* lineage tracing of labelled stem cells, intravital live cell imaging as well as the recent development of *in vitro* and *ex vivo* stem cell and sebocyte models have been beneficial for dissecting the role of distinct stem cells for SG physiology and uncovering relevant cellular and molecular mechanisms controlling sebocyte functions (45, 48–51).

### 2.2 SG formation and hair follicle morphology are closely linked

The SG forms an integral part of the pilosebaceous unit and the development of the SG is intimately connected to hair follicle morphogenesis (45, 52). As hair follicles progress through the bulbous peg stage of morphogenesis, first sebocytes emerge in their upper region (53–55). Notably, at these early stages of appendage formation, some markers of future hair follicle stem cell compartments are already expressed, including SRY-box transcription factor 9 (SOX9) as well as leucine-rich repeats and immunoglobulin-like domain protein 1 (LRIG1), molecules characteristic for the bulge and the junctional zone of the hair follicle, respectively (56–58) (**Figure 1**). During further hair follicle morphogenesis and lineage specification, SOX9+ keratinocytes migrate towards the future bulge (56, 58), whereas LRIG1+ cells are stalled in the upper part of the growing follicle structure, where sebocytes are going to emerge (58, 59). How size and localization of the different stem cell compartments are controlled during development is not understood yet. Interestingly, *SOX9* deletion impairs SG development, suggesting that SOX9+ keratinocytes affect SG formation (56). It has been shown that first sebocytes are generated in the junctional zone of the upper permanent part of the hair follicle. In particular, sebocytes emerge by asymmetric cell fate decision of proliferative Lrig1+ stem cells (58). Differentiated sebocytes are negative for LRIG1, do not proliferate and are characterized by expression of stearoyl-CoA desaturase (SCD)1 (17, 23, 45, 60, 61). Until now, the molecular mechanism driving cell fate specification within LRIG1+ stem cells during appendage formation has not been deciphered.

### 2.3 Dermal progenitor cell markers in the stroma of SG

Contribution of the dermal compartment during SG development involves wingless (*WNT*) and *bone morphogenetic protein* (*BMP*) signaling that opposingly orchestrate hair follicle and thereafter SG development (62) (**Figure 1**). More recently, an important function of the niche and extracellular matrix for regulating SG basal progenitor cells was identified. Embigin, a Wnt-regulated fibronectin receptor, binding to the N-terminal fibronectin domain without impairing integrin function increases the adhesion of basal SG cells to the extracellular matrix and regulates the expression and function of monocarboxylate ransporter (mct)1 in basal SG cells permeabilizing the cells to metabolite flow. It is specifically expressed in the SG and is promoting progression of SG basal cells towards differentiation, thus demonstrating a direct link between adhesion and sebaceous lipid metabolism. Loss of embigin leads to a SG exit from the progenitor compartment and progression toward differentiation, and compromises sebaceous lipogenesis (63).

### 2.4 Stem cells in SG homeostasis

Following the transition from SG development to homeostatic conditions in adulthood, the rate of cell proliferation declines (33, 34). Once established, SG are constantly renewed, meaning that mature sebocytes are replaced by dividing progenitor cells at the periphery of the lobes (**Figure 1**). Although the underlying molecular mechanisms governing SG cellular turnover remain to be elucidated, a recent genetic mouse model showed that individual SG lobes attached to one hair follicle are maintained by their own dedicated stem cells as one individual lobe is preserved even after ablation of the second lobe (35).

Experimental data suggest different cellular models for maintaining SG homeostasis; particularly, a role for unipotent and/or multipotent stem cells located outside and/or at the periphery of the SG was proposed (57, 64–70). The best-characterized stem cell population linked to SG renewal are LRIG1+ keratinocytes of the junctional zone of the hair follicle (57, 69). LRIG1+ keratinocytes maintain the infundibulum and junctional zone of the hair follicle and contribute to the SG during homeostasis (69). More elaborate clonal tracing studies revealed that the sebaceous duct is an integral part of the infundibulum rather that the SG (34). The differentiation of sebaceous duct cells is controlled by GATA-binding factor 6 (GATA6), a transcription factor that is upregulated in LRIG1+ progeny (71, 72). Collectively, the results point to a dual fate of LRIG1+ stem cells giving rise either to the SG or the sebaceous duct and infundibulum. The data are further supported by showing that *GATA6* depletion from the skin results in dilated sebaceous ducts and infundibulum of the hair follicle (73).

Interestingly, it has been shown that leucine-rich repeat-containing G-protein-coupled receptor 6 (LGR6)+ progenitors and keratin 15 (KER15)+ stem cells of the upper bulge/isthmus region can also generate sebocytes (68, 70, 74). In this context, LRIG1 expression was detected in KER15-derived progeny suggesting that bulge stem cells have the potential to generate SG stem cells (68).

B-lymphocyte-induced nuclear maturation protein 1 (Blimp1), another SG marker, is expressed at the base of the gland and in post-mitotic and terminally differentiated sebocytes and was shown to mark SG progenitor cells and to support terminal differentiation of sebocytes (67, 75–77). Blimp1 appears to govern the cellular input into the gland and Blimp1 depletion results in SG cell hyperproliferation (67). Depletion of Blimp1 also stimulated proliferation of bulge stem cells suggesting that perturbation of SG homeostasis affects hair follicle stem cells demonstrating a crosstalk between different stem cell compartments and a potential role for compensatory mechanisms (67, 68).

### 2.5 SG stem cells and cancer

A number of previous elaborate murine *in vivo* studies have discovered, that many different types of non-melanoma skin cancer, including sebaceous tumors originate in multipotent skin epithelial stem cells (78–80). In particular, lineage-tracing experiments demonstrated that hair follicle bulge stem cells constitute one cell of origin for mutant lymphoid enhancer-binding factor 1 (Lef1)-driven sebaceous tumors (*K14ΔNLef1* transgenic mice) (80, 81). Importantly, development of sebaceous tumors is associated with an increase in LRIG1+ stem cells indicating either a sequential cellular process of tumor initiation or formation of sebaceous tumors by different hair follicle stem cell pools (72, 80, 82, 83) (**Figure 1**). Furthermore, the sebaceous duct marker Gata6 was expressed in all sebaceous tumors developing in *K14ΔNLef1* transgenic mice (72). Depletion of *Gata6* from *K14ΔNLef1* epidermis leads to an increase in tumor incidence and frequency, suggesting that Gata6 functions as tumor suppressor in sebaceous lesions (72). Analysis of human tissues showed that expression of LRIG1 and LGR5 was present in most sebaceous carcinomas but decreased compared to benign sebaceous adenomas and expression of SOX9 was absent in sebaceous malignancies (84). Dermal progenitors, such as cellular retinoic acid-binding protein 1 (CRABP1), were expressed at the tumor-stroma interaction site within the core of sebaceous carcinomas and CRABP1 and nestin expression was lower in sebaceous carcinoma than in benign sebaceous adenomas (85).

### 2.6 Future aspects of SG developmental biology

There are still open questions to be answered, which might also explain the apparently strong SG immunological tumor protection, since SG hypertrophy and carcinogenesis are rare and mostly occur in late age (86, 87). Thereafter, the crucial signals instructing proper localization of LRIG1+ stem cells during hair follicle morphogenesis and initiating sebocyte differentiation from the LRIG1+ stem cell compartment should be elucidated (**Figure 1**). Moreover, the regulatory mode of early hair follicle stem cell specification and function and in particular, how the sebaceous duct and mature gland structures are established and what is the relationship between sebaceous duct, junctional zone and infundibulum of the hair follicle have to be clarified. At last and even more important, the lifelong maintenance of the SG lineage specification should be understood.

## 3 Bacteria and SG

### 3.1 Bacterial antigens, pattern recognition receptors and SG

The concept that acne vulgaris might be induced by a genuine inflammatory sebocyte response, first published in 2001 (88) obtained partial confirmation in 2003 through the detection of CD3+, CD4+ T cells in the perifollicular dermis of acne microcomedones before even the initiation of sebocyte and follicular keratinocyte hyperproliferation, associated with the acne lesions (89). This finding has been interpreted as an involvement of inflammatory responses in the very earliest differentiation changes of the SG and the pilosebaceous unit towards a microcomedone. Interestingly, 20 years later, an innate lymphoid cell (ILC) perifollicular distribution in wild-type C57BL/6 mouse was detected, whose immune response is dependent on factors produced by the SG (90). Indeed, sebocytes produce lymphotoxin α, which reduces sebocyte proliferation *via* Notch signaling. Reduction of the resident perifollicular infiltrate leads to sebaceous hyperplasia and seborrhea, which might alter the resident microbiome initiating an inflammatory process (91).

Human sebocytes *in vitro* constitutively express functional pattern recognition receptors, such as Toll-like receptor (TLR)2, TLR4, TLR6 and the co-receptor molecule CD14 as well as interleukin (IL)1β, IL6, IL8 and CXCL10 (92, 93). TLR play a crucial role in the induction of antimicrobial responses in various cells activating multiple steps in those inflammatory reactions that help to eliminate the invading bacteria and coordinate systemic defense. *Propionibacterium acnes* (*P. acnes*) reduces the expression of histone deacetylase 8 and 9, enhances cytokine response to TLR2 and leads to a significant amplification of the TLR agonist macrophage-activating lipopeptide 2 (MALP2)-mediated cytokine induction in human sebocytes (92). Interestingly, human acne-involved SG were shown to express common pattern recognition receptors with other inflammatory skin diseases, such as psoriasis and lichen ruber (93). Like other pattern recognition receptor modifiers, MALP2 activates not only macrophages, but all cells carrying functional TLR2/6 (94) and, therefore, also keratinocytes and sebocytes. MALP2 mainly upregulates the nuclear factor kappa-light-chain-enhancer of activated B-cells (NFκB), the transcription factors cAMP response element-binding protein (CREB), c-fos, c-jun and peroxisome proliferator-activated receptors (PPAR) through other kinases (95). The latter ones function as receptors for fatty acids (FA), especially of lipid producing cells, such as sebocytes (96, 97).

### 3.2 Bacterial biofilm and SG inflammatory response

Like many medically-relevant bacteria, also *P. acnes* forms biofilms under *in vitro*, *in vivo* and *in vivo*-like conditions. Biofilms are communities of microorganisms embedded in an extracellular matrix (consisting of self-produced extracellular polymers and host-derived material) that occur as surface-attached communities, suspended aggregates or aggregates associated with/embedded in host tissue (98, 99). Biofilm formation of *P. acnes* is particularly relevant in the context of infections related to the use of various medical devices, including prosthetic joint infections (99). Direct evidence for the involvement of *P. acnes* biofilms in acne comes from the observation that *P. acnes* biofilm-like structures are present in acne skin biopsies, and are more frequently observed in follicles of acne patients than in those of healthy controls (100–102). Detailed investigations of these biopsy samples revealed different biofilm morphologies and colonization patterns (sometimes co-occurring in the same sample), with some *P. acnes* biofilms attaching to the follicle wall or the hair shaft while in some samples biofilm aggregates were found in the lumen of the SG (101). The phenotype of biofilm-associated bacteria is very different from that of planktonic bacteria and one of the remarkable differences is the reduced susceptibility of biofilm cells to antimicrobial agents (including antibiotics and disinfectants) (103). This reduced susceptibility has also been observed in *P. acnes* biofilms and may be one of the reasons behind failure of topical and systemic antimicrobial therapy for the treatment of acne (104, 105).

While not the only player, *P. acnes* is likely to be an important contributor to the pathogenesis of acne. It proliferates in the lipid-rich and anaerobic environment of microcomedones that form as the result of increased sebum production and hyperkeratinization of keratinocytes, and this proliferation activates immunocompetent keratinocytes and sebocytes (106). *P. acnes* produces many factors that contribute to pathogenesis (104), including lipases (that hydrolyze sebum triglycerides to free FA which in turn act as damage associated molecular patterns) (107) and co-hemolytic Christie–Atkins–Munch-Peterson (CAMP) factors and porphyrins. Combined these virulence factors induce a pro-inflammatory response in human keratinocytes and sebocytes. This pro-inflammatory response includes the production of IL1β due to activation of the NLRP3 inflammasome (91, 108). While most of the studies on production of virulence factors have been carried out *in vitro*, there is evidence that production of specific virulence factors is typically higher in biofilm-grown *P. acnes* than in planktonic cultures, but also that virulence factor production can be strain-dependent (104, 109–112). Recent work in an *in vitro* co-culture model, in which HaCaT keratinocytes and SZ95 sebocytes were combined in a ‘well and insert’ system together with *P. acnes* biofilm aggregates (113, 114), confirmed that biofilms formed by different *P. acnes* strains interacted differently with the human host. Indeed, compared to type II strains, acne-associated type I strains showed a higher association to both HaCaT keratinocytes and SZ95 sebocytes, and resulted in higher breakdown of tight junctions; in contrast, the invasion frequency was higher in case of type II strains (114). Importantly, different *P. acnes* strains also elicit different inflammatory responses. Acne-associated *P. acnes* strains activate the NRLP3 inflammasome assembly, which leads to increased IL1β production, something not observed in strains that are not associated with acne. The same increased IL1β production was also observed when porphyrin extracts of acne-associated *P. acnes* were used. In agreement with this observation, it was found that *P. acnes* strains associated with acne produce higher levels of porphyrins and this high porphyrin production leads to activation of the inflammasome *via* the induction of K^+^ leakage (113).

Although much remains to be learned about the role of *P. acnes* biofilm formation in the pathogenesis of acne, the available evidence strongly suggests that biofilm formation is relevant *in vivo* and that *P. acnes* biofilms and/or compounds produced by biofilm cells contribute to the inflammatory response observed.

## 4 Immunology of the SG

### 4.1 Innate defense mechanisms of the SG

**Figure 2:**
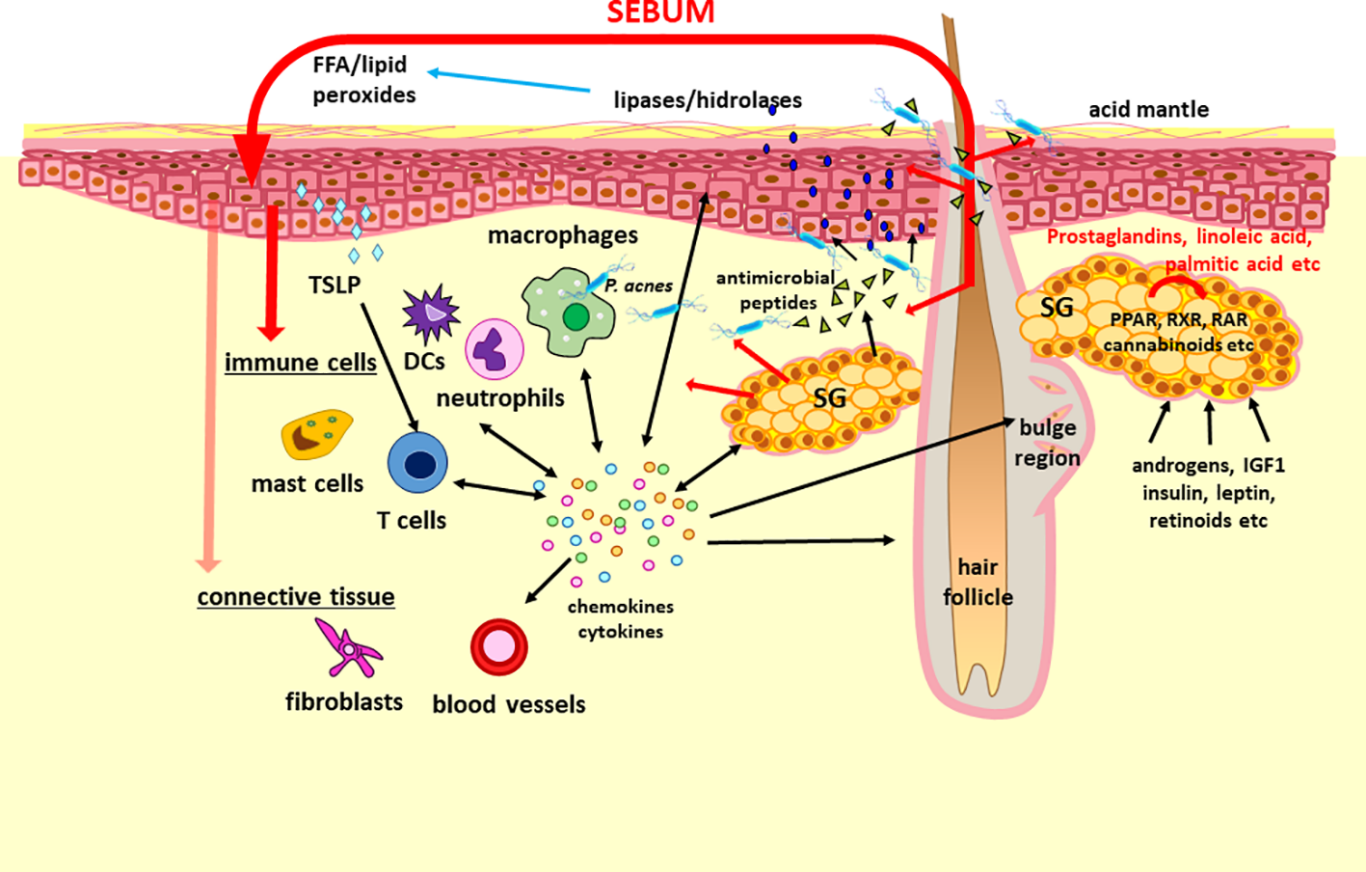
The concept of “sebaceous immunobiology”. Sebum production and composition is regulated by intrinsic (i.e. activators of PPAR and RXR nuclear receptors such as prostaglandins, linoleic acid and endocannabinoids) and extrinsic (i.e. androgens, IGF1, insulin, leptin and retinoic acid) factors. Although the primary role of the lipid rich sebum is to lubricate the hair and to contribute to the lipid barrier of the skin, the composing lipids may penetrate also into the dermis (marked with red arrows). Therefore, they may modulate the functions of keratinocytes, immune cells and presumably of stromal cells by altering their gene and protein expression. Importantly, sebocytes are also able not only to respond to but also to produce a large number of cytokines and chemokines which may be important in maintaining the physiological dermal immune milieu such as the Th17 signature of the sebaceous gland rich skin or hair growth. Regarding its interactions with the microbiota, sebum lipids exert antimicrobial effects, may modulate the macrophage – P. acnes interaction, but are also converted by various microbes into lipids with inflammatory properties, which could have a role not just in the pathogenesis of acne but also in other diseases. These altogether support that a change in the amount and composition of the sebum could modulate (patho)physiological settings and thus may be therapeutic target as well. DCs, dendritic cells; FFA, free fatty acids; IDF1, insulin-like growth factor 1; PPAR, peroxisome proliferator-activated receptors; RAR, retinoic acid receptors; RXR, retinoid X receptors; SG, sebaceous gland; TSLP, thymic stromal lymphopoietin.

Although the innate immune response includes physical, chemical and cellular defenses, the latter mediated by a variety of immune cells such as natural killer cells, macrophages, neutrophils, dendritic cells, mast cells, basophils, and eosinophils, more and more data support that cells of non-myeloid or lymphoid origin may also be immunologically competent in the skin. After extensive studies on keratinocytes (115–117), sebocytes are now also accepted as potent immune regulators, integrating into the innate (non-specific) immune responses of the skin (**Figure 2**). Expressing numerous cytokines and other inflammatory mediators (108, 118, 119), thus potentially modulating the activity of immune cells, sebocytes may contribute also to the polarization of cutaneous T cells towards the Th17 phenotype, a subset of Th cells that are particularly important in skin homeostasis and inflammation (22). Conversely, sebaceous activity in humans and mice are influenced by ILC and also by T cells, as well by cytokines such as thymic stromal lymphopoietin (TSLP), tumor necrosis factor (TNF)α, IL4 and IL13 (25, 90, 120).

Being at a unique “locus minoris resistenciae” within the dermis, sebocytes play an important role in conserving and balancing the symbiotic environment of lipids, cytokines and resident bacteria of the pilosebaceous unit, in which their production of antimicrobial peptides (AMP) takes a central position (**Figure 2**). AMP are evolutionarily ancient innate immune effectors that support tissues in coping with microbial challenges, as they rapidly kill or inactivate microorganisms. In particular, the cells lining the gut, skin and respiratory tract produce a wide variety of AMP, which makes sense in light of the microbial load faced by these tissues (121). AMP vary greatly in their target organism specificity and mechanism of action, most frequently killing bacteria by targeting cell wall or cell membrane structures (121).

Mammalian AMP are grouped into various protein families, such as β-defensins, cathelicidins, resistin, and S100 proteins. Importantly, several members of these families have been shown to be expressed by SG and to exert antimicrobial activity. While expression of AMP such as β-defensin (hBD)1 and 2 (122) and psoriasin (123–125) in SG *in situ* and in cultured SZ95 sebocytes (108, 125, 126) was known for years, the first functional confirmation of antimicrobial activity of a sebocyte-secreted peptide was reported for cathelicidin (127) and for histone H4 (128). In particular, both sebocyte-extracted and recombinant histone H4 showed antimicrobial activity against *Staphylococcus aureus* (*S. aureus*) (128). Cathelicidin (LL-37) killed both *S. aureus* and *P. acnes*, but was found to be present at rather negligible amounts in sebocyte extracts; further studies suggested that combination with other AMP, such as with psoriasin, greatly increases its antimicrobial potency (127). Protease-activated receptor 2 (PAR2), a multifunctional membrane receptor that regulates production of certain AMP (129), and dermcidin, a peptide usually thought to be sweat-gland specific (130) have also been detected in SG. More recently, expression of resistin-like molecule a (RELMa) by epidermal keratinocytes and sebocytes and its role as an AMP was reported: RELMa expression was induced by microbiota colonization of mouse skin, it was found to be bactericidal *in vitro*, and protected against bacterial infection of the skin *in vivo* (131). Finally, small proline-rich proteins (SPRR) were identified in SG, and displayed potent bactericidal activity against methicillin-resistant *S. aureus*, *Pseudomonas aeruginosa*, and skin commensals (132).

### 4.2 Cross-regulation of SG and immune cells

Immune cells are found in the upper region of the hair follicle, the area surrounding the SG, in the steady state, and accumulation of immune cells into the upper follicular area is observed during inflammation. The recruitment and tissue homeostasis of immune cells are regulated by chemokines and cytokines produced by hair follicle and/or SG. In other words, pilosebaceous units work as immunological hubs in the skin (133). For example, repopulation of Langerhans cells into epidermis is guided by chemokines produced by hair follicles (134), resident memory T cells are maintained in steady state by hair follicle-producing IL7 and IL15 (135), hair follicle-derived CCL20 regulate neonatal migration of regulatory T cells (Treg) (136). In recent years, the mechanisms by which immune cells in perifollicular areas actively regulate SG function have been revealed (**Figure 2**).

TSLP is produced by, among others, fibroblasts and is a cytokine that drives Th2 immune response. Interestingly, TSLP promotes systemic lipolysis by activating T cells and stimulating the secretion of sebum from SG. Systemic expression of TSLP using adeno-associated virus led to T cell-dependent decrease in white adipose tissue, which was associated with the accumulation of T cells around SG and increased secretion of sebum. Furthermore, TSLP deficiency at steady state led to decreased production of sebum and antimicrobial peptides, suggesting that physiologic levels of TSLP regulate homeostatic sebum production and skin barrier function. This study demonstrated the existence of a mechanism by which sebum secretion-dependent energy expenditure plays a role in the regulation of systemic metabolism (120). On the other hand, how TSLP-induced T cells stimulate holocrine secretion in SG has not been identified yet, and cytokines or other soluble factors produced by T cells also remain to be elucidated. For example, the characteristic Th2 cytokines IL4 and IL13 increase sex hormone synthesis in the skin by stimulating the expression of 3β-hydroxysteroid dehydrogenase (HSD)1, a key rate-limiting enzyme in sex steroid hormone synthesis in SG (25). Thereafter immune cell-produced cytokines affect various aspects of SG function. It is noteworthy that TSLP expression is higher in SG-rich area of human skin, and T cells and dendritic cells are found in greater numbers than in SG-poor areas (119). Although it is not known how these cells are recruited around SG and how TSLP expression is induced in SG-rich areas, the viewpoint of cross-regulation formed between TSLP, T cells, and SG will provide new insights into the mechanisms involved in SG-mediated lipid metabolism and the pathogenesis of inflammatory skin diseases.

It is reasonable to assume that the regulation of SG function by immune cells is antigen independent. Therefore, it is likely that innate immune cells play a cooperative role with SG. ILC are innate counterparts of helper T cells. They lack T-cell receptors and are directly activated by cytokines such as IL33, IL25, and TSLP produced by other cell types. In other words, the effector function of ILC does not depend on antigens. There are three groups of ILC, namely ILC1, ILC2, and ILC3, which are defined by transcription factors and effector cytokines (137). These are equivalent of Th1, Th2, and Th17 in helper T cell subsets. ILC are tissue-resident immune cells with tissue-specific phenotypes and functions, and engage in tight cross-regulation with surrounding mesenchymal cells. In recent years, it has become clear that ILC with diverse phenotypes and functions exist in the skin (138, 139). Of particular interest is the ILC in the upper region of the hair follicle, at the base of SG, where ILC maintain the balance of the skin microbiota by negatively regulating SG function. These ILC located at epidermis are maintained by IL7 and TSLP, and their tissue distribution is regulated by C-C chemokine receptor type 6 (CCR6). *Rag2* ^-/-^ *Il2rg* ^-/-^ mice lacking ILC displayed SG hyperplasia which resulted in excessive antimicrobial lipid secretion and imbalance of the skin microbiota. ILC suppressed Notch signaling in sebocytes by producing lymphotoxins (90). Notch signaling is an important canonical pathway for cell proliferation and differentiation in hair follicles and SG (35), and Notch ligands produced by Tregs are known to promote differentiation of hair follicle stem cells (140). Crosstalk between immune cells and pilosebaceous units *via* Notch signaling may be an important factor in skin barrier homeostasis.

### 4.3 Integrating sebaceous lipids into “sebaceous immunobiology”

Substantial evidence supports that the (patho)physiological role of sebocyte-derived lipids exceeds their primary function to lubricate the hair (141). The findings that epidermal (keratinocyte-derived) lipids make up a minor fraction of total surface lipids on areas rich in SG (142) suggests that sebum lipids also contribute to the construction of the lipid barrier (**Figure 2**). Importantly, these lipids may play a role not only as moisturizers but also regulators of the microbiota, e.g. lauric and sapienic acid as well as long-chain bases (sphingosine, dihydrosphingosine and 6-hydroxysphingosine) have broad-acting antimicrobial properties (143), providing a partial explanation for the observed differences in the microbiota of various topographical regions of the human body (144). It is worth noting, that AMP and sebaceous lipids, while showing distinct chemical structure and modes of action, seem to cooperate to provide the best possible protection: the antimicrobial action of sebum lipids was enhanced by histone H4 (128), while free FA can induce sebocytes to upregulate expression of hBD2 (126).

Importantly, sebocyte-derived lipids are also able to penetrate into the skin, and therefore, may alter not only the gene expression of keratinocytes but might also modulate the maturation and immune responses of immune cells such as macrophages (19, 21, 118) (**Figure 2**). Each of the sebum composing lipids had a different effect on the polarization of macrophages and their interaction with *P. acnes*. Notably, *in vitro* studies found that linoleic acid and oleic acid were potent inducers of the alternative activation of macrophages involved in tissue homeostasis and repair functions. Moreover these lipids also enhanced the phagocytic capacity of macrophages towards *P. acnes*. Linoleic acid had also an anti-inflammatory effect in *P. acnes*-activated monocyte-derived macrophages by inhibiting the secretion of IL1β (19). Delivering further important findings on the immune modulatory effects of the secreted sebum lipids on sebocytes themselves, palmitic acid may be a potent pro-inflammatory stimulus also for sebocytes under certain conditions such as the presence of epidermal growth factor (EGF) (20, 145, 146). Sebocyte-derived proteins and lipids may modify the microbiota through nutritional as well as anti-microbial actions. Moreover, sebum lipids contribute to the lipid milieu of the dermis, which may regulate the (immune) phenotype of various cells. Finally, in response to various stimuli, sebocytes are able to change their lipid production as well as their inflammatory gene expression and protein profile. Taken together, the above data led to the introduction of the concept of “sebaceous immunobiology” (21) (**Figure 2**).

The *in vivo* relevance of “sebaceous immunobiology” was supported by the cutting edge finding that prominent immune-topographical differences exist between SG-rich and poor skin. Besides the increased amount of lipids, the increased chemokine (CCL2, 3, 19, 20, 23, 24) and antimicrobial peptide (S100A7, A8, A9, lipocalin, hBD2) expression, altered barrier (keratin 17, 79) functions, and a non-inflammatory Th17/IL17 dominance was detected that altogether marked a characteristic innate and adaptive immune and barrier milieu in the SG-rich regions of the body (147, 148).

Regarding pathological settings, “sebaceous immunobiology” led to a change in our current understanding of acne pathogenesis as well (149). While the contribution of the increased amount and altered composition of sebum to the *P. acnes* dysbiosis is unquestionable, we proposed that the change in sebum production may itself be a potent inducer of inflammation targeting the aforementioned immune cells and keratinocytes (150). Indeed, in case of *in vitro* differentiated, monocyte-derived macrophages, squalene, palmitic acid, stearic acid and oleic acid augmented the secretion of IL1β, even in the absence of *P. acnes*, whereas oleic acid had a selective effect of inducing IL1β, but downregulating IL6 and TNFα secretion (19). Therefore, the potential in sebosuppression and sebum modulation may be far more complex than modifying the biofilm formation, as it could directly modulate the function of inflammatory cells.

Finally, it is important to note that the challenging, new concept of “sebaceous immunobiology” is not restricted to the pathogenesis of acne. Indeed, whether a change in the density/activity of SG may have a pathological relevance in diseases such as seborrheic dermatitis, rosacea, atopic dermatitis, psoriasis and hidradenitis suppurativa or could explain the predilection sites of certain inflammatory diseases awaits to be answered. Likewise, it should also be systematically investigated whether sebum-modifying therapies could be applied to other (non-acne) inflammatory skin diseases.

## 5 Endocrine molecules and SG inflammation

### 5.1 *In situ* production and action of sex steroids in human SG physiology and pathology

**Figure 3:**
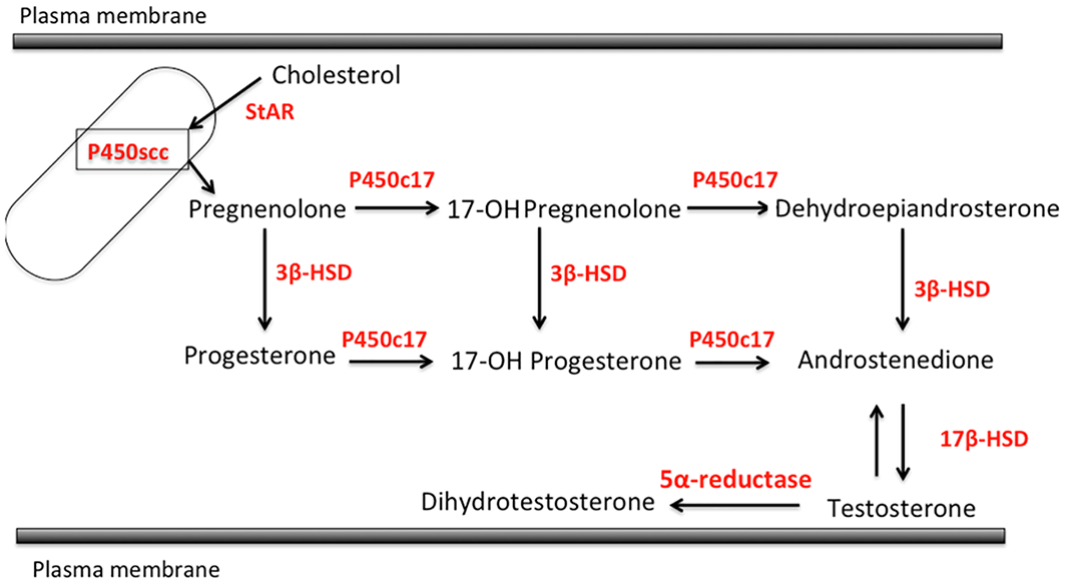
Steroidogenic pathways relevant to human sebaceous gland (StAR, steroidogenic acute regulatory protein; P450sc, P450 side chain cleavage; 3β-HSD, 3β-hydroxysteroid dehydrogenase; P450c17, cytochrome P450c17; 17β-HSD, 17β-hydroxysteroid dehydrogenase).

**Figure 4:**
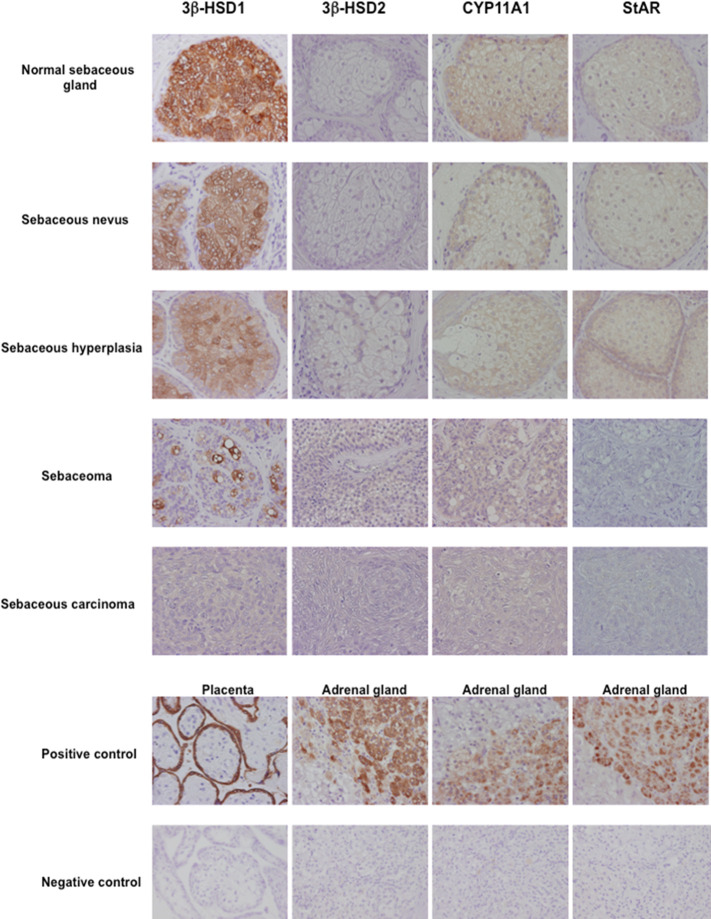
Representative illustration of 3β-hydroxysteroid dehydrogenase (3β-HSD) 1, 3β-HSD2, cytochrome P45011A1 (CYP11A1) and steroidogenic acute regulatory protein (StAR) in human sebaceous gland and its disorders. Marked cytoplasmic 3β-HSD1, CYP11A1 and StAR immunoreactivity was abundant in normal sebaceous glands and weak in pathological sebaceous glands (magnification 200x).

**Figure 5:**
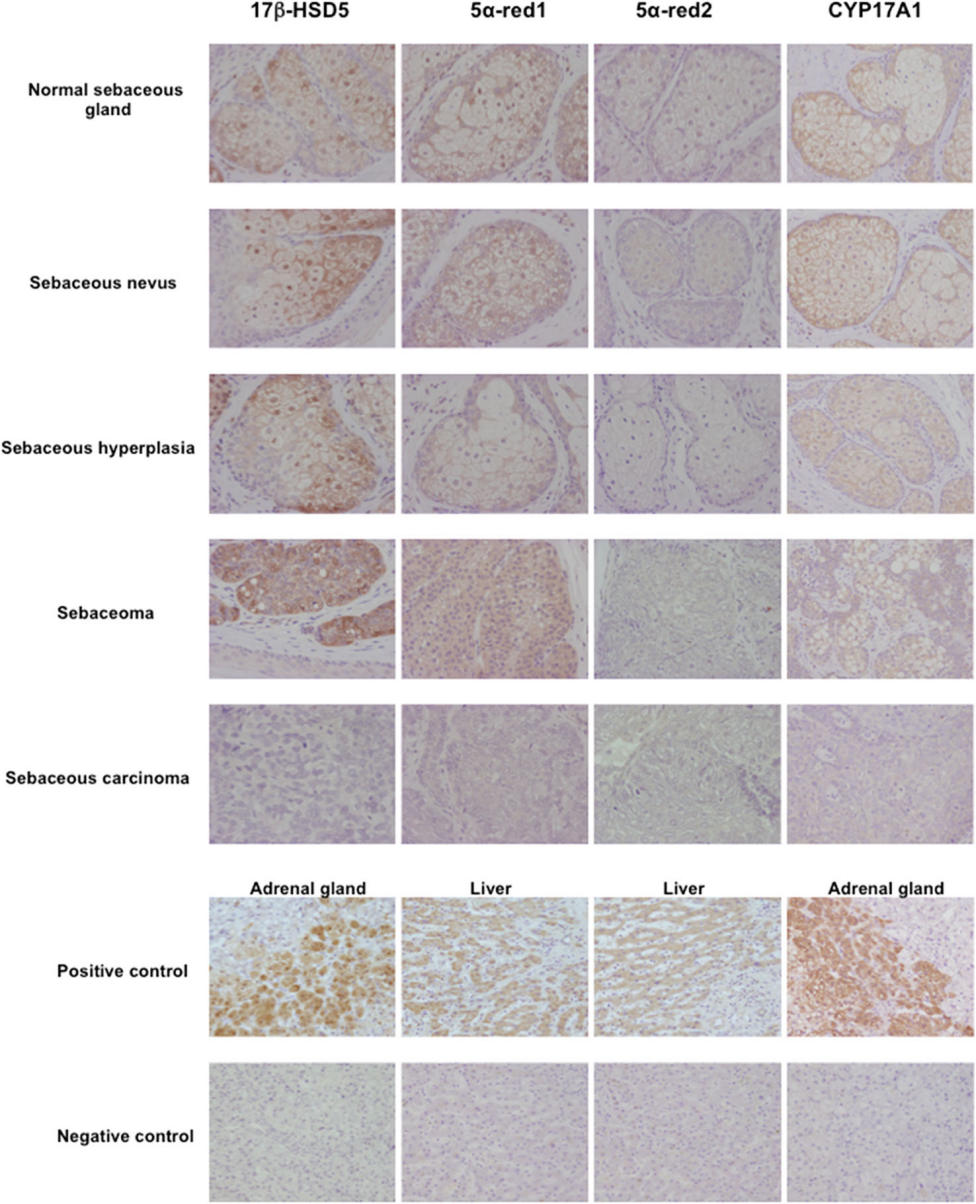
Representative illustration of 17β-hydroxysteroid dehydrogenase 5 (17β-HSD5), 5α-reductase (5α-red)1, 5α-red2 and cytochrome P45017A1 (CYP17A1) immunoreactivity in human sebaceous gland and its disorders.17β-HSD5, 5α-red1 and CYP17A1 immunoreactivity was predominantly detected in normal sebaceous glands compared to pathological sebaceous glands (magnification 200x).

Skin is well known as an important site of synthesis and metabolism of sex steroids (151, 152) (**Figure 3**). Importantly, SG are among those components, whose functions are influenced by sex steroids. Results of several previous clinical and experimental studies of SG also indicated the potential roles of alterations in steroid hormone metabolism in their disorders (153). Steroidogenic enzymes, including steroidogenic acute regulatory protein (StAR), Cytochrome P450 (CYP)11A1, 3β-HSD, 17β-HSD, CYP17A1 and 5α-reductase 1 and 2 have all been detected in normal human SG (5, 154–157) (**Figures 3**-**5**). Those enzymes were also reported in their pathological conditions such as SG hyperplasia, adenoma and carcinoma (9, 153, 158) (**Figures 4**, **5**). Among those steroidogenic enzymes above, 3β-HSD is the pivotal enzyme in the cascade of androgen production. Azmahani et al. (9, 158) demonstrated the presence of 3β-HSD1 and the absence of 3β-HSD2 in human SG under both normal and pathological conditions (**Figures 4**, **5**). This differentiation of 3β-HSD subtype is considered important, as type 1 and type 2 distinctively differ in the human SG, suggesting that different transcription factors could possibly regulate the transcription of each isoform, and this regulation could be also associated with a tissue/cell-specific pattern. In addition, the decreased expression steroidogenic enzymes from sebaceous nevus to carcinoma through sebaceous hyperplasia and sebaceoma could also serve as a possible underlying factor in the development of these conditions (9, 158) (**Figures 4**, **5**).

Both androgen receptor and progesterone receptor (PR) are present in normal sebocytes (159). In addition, androgen receptors were detected in basal and differentiated sebocytes, indicating that androgens are primarily involved in the regulation of cell proliferation and lipogenesis of SG (160). These hormone receptors were also reported in various disorders of human SG including hyperplasia, adenoma and carcinoma (9, 158). Acne and other relatively uncommon sebaceous disorders including sebaceous hyperplasia, sebaceoma and carcinoma are truly androgen-related disorders and the inhibition of the formation and action of androgens is reasonably postulated to be a logical and effective way to treat these diseases. Therefore, in addition to the classical steroidogenic tissue, SG possess the enzymatic systems required for the formation of the active androgens (5, 159). On the other hand, estrogen receptor (ER)α and ERβ were also reported in normal human sebocytes but significantly lower in SG tumors than normal SG (161).

The above findings did indicate that pathogenesis of human SG-related disorders and neoplasms, similarly to certain forms of breast cancers, may include intracrine mechanisms, e.g. aberrant *in situ* production of sex steroids. The presence of steroidogenic enzymes as well as androgen receptors and PR in those cells demonstrated that SG and its disorders could produce active sex steroid hormones *in situ* from precursor or biologically inactive steroids in the circulation and these locally produced biologically active sex steroids, primarily androgens, could act on the cells through their receptor. Of particular importance, this intracrine mechanism in SG and its disorders makes it possible to proceed various androgen-related features of the gland, independent of circulating levels of biologically active sex steroid hormones.

Therefore, it has become important to explore regulatory mechanisms of expression of those steroidogenic enzymes involved in local or *in situ* sex steroid production such as their transcription factors in human SG. Azmahani et al. (9, 158) demonstrated the expression of several transcription factors and orphan nuclear receptors known to regulate the expression of steroidogenic enzymes, including nuclear steroid hormone receptor (NGFI-B) and GATA-binding factor 6 (GATA6). Their transcriptional regulation of the StAR, CYP11A1 and 3β-HSD has been also reported in the adrenal gland, testis, ovary and placenta (162, 163). In particular, NGFI-B was reported to regulate the transcription of StAR, CYP11A1, CYP17 and 3β-HSD2 in ovarian theca cells (164). Therefore, the transcription factors NGFI-B and GATA6 could modulate the levels of StAR, CYP11A1 and 3β-HSD1 in human SG. Further investigations are needed to clarify the potential involvement of local and systemic factors in the regulation of those transcription factors.

### 5.2 PPAR and SG function

The nuclear receptor PPAR are ligand-activated transcription factors that modulate the expression of multiple genes involved in the regulation of lipid, glucose and amino acid metabolism. PPAR and cognate ligands also regulate important cellular functions, including cell proliferation and differentiation as well as inflammatory responses (165). In humans all three PPAR isotypes are expressed in epidermis and PPARγ is found mostly in the more differentiated suprabasal keratinocytes (96). This isotype is also abundantly expressed in skin adipocytes and plays a critical role in adipocyte differentiation. Human SG, express all known PPAR isoforms. However, PPARγ is the main functional isoform of the PPAR family members expressed in the normal SG and cultured sebocytes, correlating with their more differentiated state (166, 167).

In the nucleus, PPARγ forms a heterodimer with the nuclear receptor retinoid X receptor (RXR) to bind to genomic DNA at specific sites. Ligand independent repression, agonist-dependent activation and antagonist-dependent repression are the three major mechanism of PPARγ modulation like other nuclear receptors. PPARγ interacts *via* its ligand-binding domain with many structurally different small molecules. PPARγ-based gene regulation comprises both gene activation and repression events, depending on the tissue and its molecular context. PPARγ coactivator 1 (PGC1) or corepressing proteins, such as nuclear receptor corepressor 1 (NCoR1), can assemble with PPARγ and modify its activity. The protein complexes are probably regulated by several post-translational modifications such as phosphorylation, acetylation, glycosylation, SUMOylation, and ubiquitination (165).

Physiological endogenous ligands that activate PPARγ include FA, such as linoleic acid, various eicosanoids, and other lipids present in the skin and in SG. PPARγ has a relevant role in controlling differentiation and lipid metabolism in sebocyte-like cells of rat preputial glands, isolated human SG and cultured human sebocytes. PPARγ has been shown to increase lipid accumulation in rat preputial sebocytes and in human sebocytes, and patients treated with thiazolidinediones, a class of PPARγ agonists, experienced increased sebum production (168, 169). Experimental models using SZ95 sebocytes have demonstrated that arachidonic acid and arachidonic acid keto-metabolites, e.g. 5-oxo-6E,8Z,11Z,14Z-eicosatetraenoic acid (5-KETE), 12-oxo-5Z,8Z,10E,14Z-eicosatetraenoic acid (12-KETE), regulate PPARγ signaling pathways modulating phospholipid biosynthesis and inducing neutral lipid synthesis (96, 97, 118, 167).

PPARγ is expressed in suprabasal and differentiating sebocytes, correlating with the differentiation level and specifically with the expression of Ki67 and p63, markers of cell cycle activity and differentiation. Costaining of Ki67 and PPARγ protein gave indication of an inverse relationship. In SG, PPARγ expression correlates with that of the androgen receptor and serves as the cofactor required for the display of androgens effects on sebocytes. In differentiating adipocytes, PPARγ induces degradation of β-catenin and inhibits WNT signaling, thereby resulting in their differentiation. Since low levels of WNT signaling promote sebaceous differentiation, it is likely that PPARγ suppresses WNT signaling as part of the sebocyte differentiation program. In sebaceous hyperplasia, the expressions of PPARγ is equal to normal SG. The proportion of cells expressing PPARγ is lower in sebaceous adenoma and carcinoma, whereas those of Ki67 and p63-positive cells is higher (97, 118, 167, 169).

Anti-inflammatory effects of ligand-modulated PPARγ seem to involve interconnected mechanisms. Studies with different agonists have demonstrated several mechanisms involved including protein–protein interactions, interaction with NFκB or cofactor competition of both transcription factors. Moreover, regulation of protein localization and post-translational modifications including ubiquitination of PPARγ and subsequent degradation of NFκB have been shown. Protein–DNA binding events can also be influenced by SUMOylation of PPARγ, leading to transrepression of NFκB or by other competitive binding events. PPARγ agonists exert an anti-inflammatory effect partly dependent on the ability to antagonize other transcription factors, such as members of the NFκB and activator protein-1 (AP1) families, having a key role in inflammatory processes. Moreover, they can counteract the degradation of IκBα and increases its protein expression, thus preventing the nuclear translocation of NFκB (2, 97, 167, 170, 171). Being able to modulate sebaceous lipogenesis, differentiation and anti-inflammatory response, PPARγ has represented a candidate for understanding the correlation between altered lipid synthesis and generation of inflammation in sebum-dependent inflammatory skin disease such as acne and possibly seborrheic dermatitis.

PPARγ and its target genes adipose differentiation-related protein gene (*ADRP*), encoding adipophilin, and angiopoietin-related gene (*PGAR*), which regulates lipogenic pathways and sebaceous lipogenesis at protein and mRNA level, are expressed at lower levels in SG in both involved and non-involved skin from acne patients associated with the increase in endogenous lipid ligands, such as eicosanoids. The basal level expression of genes and proteins involved in insulin-/insulin-like growth factor 1 (IGF1)-induced lipogenesis, proliferation and inflammation are significantly higher in sebocytes with reduced or abolished PPARγ expression, suggesting the involvement of this nuclear receptor in the control of cellular physiological processes (167, 170).

In affected and non-affected acne skin lower expression of PPARγ and hyper-activation of mechanistic target of rapamycin (mTOR) signaling have been detected (167). *In vitro*, less differentiated sebocytes exhibit low level of PPARγ associated with high expression of insulin receptor and an up-regulation of mTOR pathway leading to altered lipogenesis. The treatment with the selective PPARγ modulator N-acetyl-GED-0507-34-LEVO (NAC-GED; GMG-43AC) promoted sebocyte differentiation, decreased insulin receptor expression and inhibited mTOR pathway, leading to a significant decrease in the effects of insulin challenge on sebogenesis and inflammatory response. *In vivo* the effect of the topical treatment with NAC-GED was evaluated by analyzing the protein expression in the collected sebum from the treated patients. Due to the fact that sebocyte death is followed by holocrine secretion, proteins generated inside the sebocyte are excreted together the lipid component. Associated with the improvement of the skin manifestations, increased PPARγ levels associated with decreased expression of mTOR target proteins, such as hosphoi-S6, and of lipogenic genes, such as desaturase enzymes, were detected in patients’ sebum. Moreover, a significant decrease of inflammatory cytokines, such as IL6 and IL8, and a qualitative normalization of the sebum composition with a decreased level of the monounsaturated FA sapienic acid were observed. The reduction of hyperkeratinization, mirrored by the decrease of comedones, accounts for PPARγ-mediated modulation of proliferation and differentiation of keratinocytes (172). The *in vivo* results validated the *in vitro* data reinforcing the idea of a strict correlation between altered sebogenesis and generation of inflammatory process from sebocytes and that their altered differentiation process can be the basis for the appearance of acne (173). More recently, a phase 2 trial has confirmed the clinical efficacy of the molecule (174).

Altogether the available data clearly indicate that PPARγ has multiple physiological roles in sebocytes. It contributes to proper differentiation process, appropriate sebogenesis and regulation of the inflammatory processes. Therefore, PPAR in general, and the γ isoform in particular, are relevant therapeutic targets for certain SG-related inflammatory skin diseases and its appropriate modulation appears to contribute in restoring “normal” metabolic activity of the cells.

### 5.3 Neuropeptides and SG immunity

SG are equipped by rich vascularization and microcirculation (4), while their innervation remains controversial. A network of nerve fibers encircles the hair follicle and is tangent to the SG. However, nerve fibers entering the SG have only been convincingly demonstrated in association with acne-involved SG (175). Interestingly, the human skin and in particular the SG express functional receptors for neuropeptides (NP), a heterogeneous group of biologically active peptides that are present in neurons of both the central and peripheral nervous systems, such as CRH, melanocortins, β-endorphin, vasoactive intestinal polypeptide, neuropeptide Y, and calcitonin gene-related peptide (159, 176). Circadian secretion of CRH from the hypothalamus affects the pituitary gland. The latter synthesizes proopiomelanocortin (POMC) and decomposes it into several biologically active substances, e.g. corticotropin (ACTH), β-endorphin, α-melanocyte-stimulating hormone (α-MSH; melanocortin) (176).

**Figure 6:**
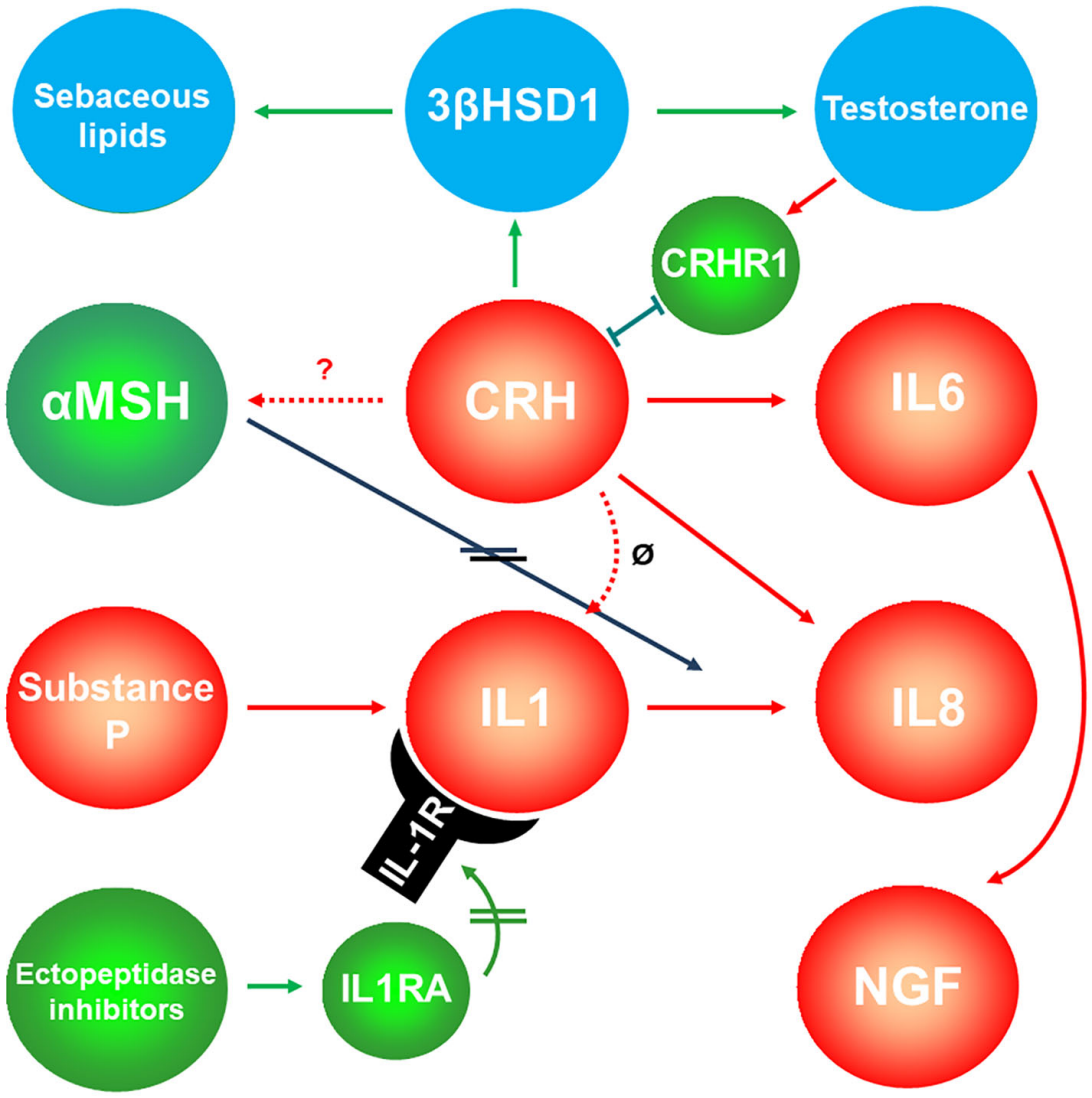
Interaction of sebaceous gland-expressed neuropeptides and sexual hormones. Corticotropin-releasing hormone (CRH) – after binding the corticotropin-releasing hormone receptor 1 (CRHR1) -stimulates sebaceous lipogenesis and testosterone release, while testosterone inhibits the expression of CRHR1. Green symbols and arrows indicate anti-inflammatory activity; red symbols and arrows indicate inflammatory potential. Double lines on the arrows indicate inhibition. Blue symbols indicate targets of the pathway, whereas testosterone might induce a negative feedback to CRH by inhibiting the expression levels of its receptor CRHR1. ø, CRH does probably not inhibit IL1. 3βHSD1, 3β-hydroxysteroid dehydrogensase 1; αMSH, α-melanocortin-stimulating hormone; IL1RA, Interleukin 1 receptor antagonist; NGF, neural growth factor.

Activation of the CRH receptor 1 (CRHR1) affects immune and inflammatory processes and is involved in the development and the stress-induced exacerbation of acne. CRH-binding protein (CRHBP) has a buffering role in response to the stress attack in acne by serving as a negative regulator of local CRH availability (177). On the other hand, CRHR2 exhibits the most significant expression within SG and possibly regulates local SG functions by having a direct influence on sebum production (6). In addition, CRH significantly induces sebaceous lipogenesis, IL6 and IL8 secretion and mRNA levels of 3β-HSD/Δ^5–4^ isomerase (6, 178) (**Figure 6**).

α-MSH has been identified as sebotropin, pigmentation hormone and modulator of inflammatory and immune tissue responses within the pilosebaceous unit (179–181). The effects of α-MSH are mediated *via* binding to melanocortin receptors (MCR), which belong to the superfamily of G protein-coupled receptors. The presence of both MC1R and MC5R was detected in primary cell cultures of facial human sebocytes and in SZ95 sebocytes. The expression of MC5R is weaker than that of MC1R, but it has been shown to be a marker of human sebocyte differentiation, since its expression increases in lipid-accumulating sebocytes (181, 182). As proinflammatory cytokines are upregulated in acne lesions, sebocytes may respond to these signals with increased MC1R expression, thereby generating a negative feedback mechanism for αMSH, which exerts direct anti-inflammatory actions, i.e. inhibition of IL1α-induced IL8 secretion (180, 181). The activation of the lipid-mediated pathway through PPAR could subtend the capacity of αMSH to influence cell proliferation and differentiation in sebocytes and melanocytes as well as lipogenesis and the inflammatory process in sebocytes. Moreover, variation of MC1R expression may be associated with different rates of sebogenesis in the different skin phototypes (183).

Substance P, expressed in small nerves around the acne-involved SG (175, 184), promotes the development of cytoplasmic organelles in sebocytes, stimulates proliferation, and induces a significant increase of sebocyte size and SG volume. Substance P expression has also been associated with increased innervation around the acne-involved SG (175). The latter is related to increased expression of nerve growth factor in acne-prone SG. The abundant IL6 expression in inflammatory SG is directly regulated by nerve growth factor (184). Neutral endopeptidase, a peptidase that degrades substance P, is highly expressed in the SG of acne patients (175).

These findings indicate an active involvement of the hypothalamus-pituitary-adrenal axis in the modulation of SG immunity but also the existence of an active local neuroimmunological network of the SG that may directly influence skin inflammatory processes.

### 5.4 Cannabinoids and inflammation in SG - A “high”-way to heal?

#### 5.4.1 Introduction to the complex (endo)cannabinoid signaling

Cannabinoid signaling is an important regulator of several physiological processes (185, 186). Although its best studied functions are related to the central nervous system, one should keep in mind that members of the endocannabinoid system (ECS) can be found at the periphery on multiple cell types including, but not limited to various elements of the immune system and the skin (186–191). Indeed, without being exhaustive, the endocannabinoid tone, and especially activation of the major cannabinoid receptors CB_1_ and CB_2_ were shown to exert mostly anti-inflammatory/immunosuppressive effects in various systems and cell types (186, 187, 191, 192).

**Figure 7:**
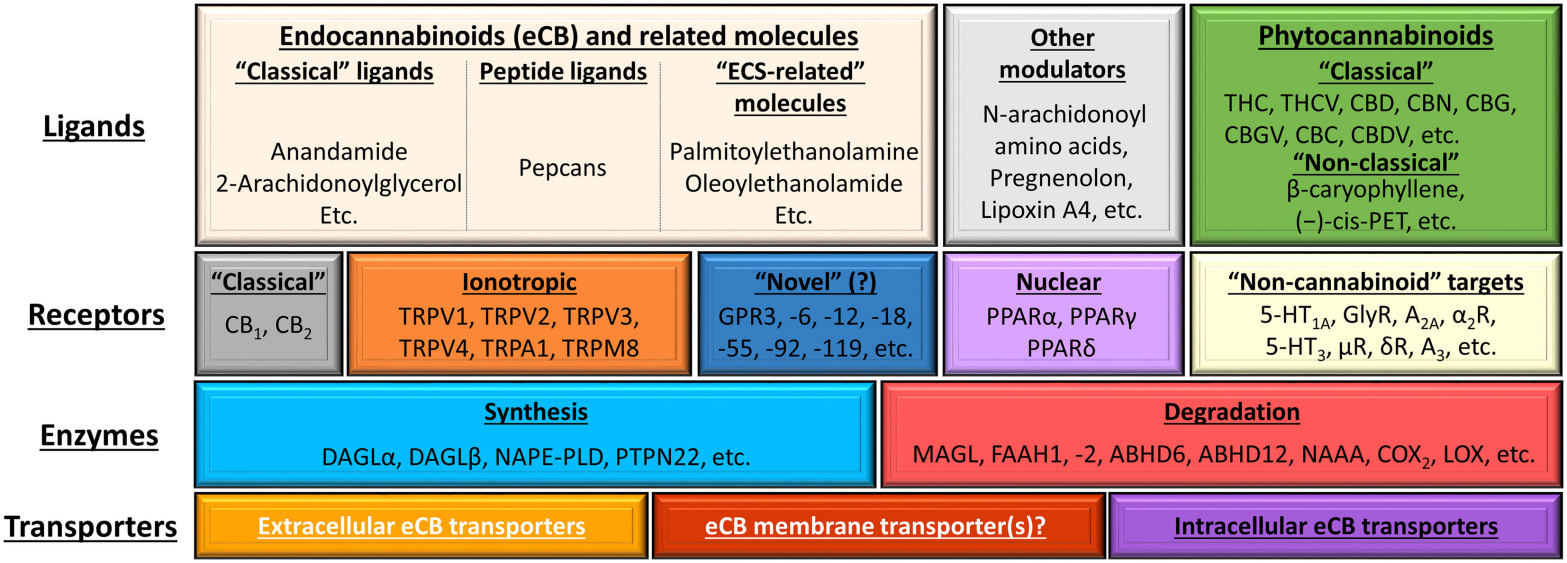
Overview of the (endo)cannabinoid system and its putative connections to other signaling systems. The figure was adapted and modified from (190) originally licensed under CC-BY, version 4.0. 5-HT, 5-hydroxytryptamine (serotonin) receptor; A_2A_ and A_3_, adenosine 2A and 3 receptors; ABDH6 and -12, α/β-hydrolase domain containing 6 and 12; CBC, (-)-cannabichromene; CBD, (-)-cannabidiol; CBDV, (-)-cannabidivarin; CBG, (-)-cannabigerol; CBGV, (-)-cannabigerovarin; CBN, (-)-cannabinol; (-)-cis-PET (–),-cis-perrottetinene; COX_2_, cyclooxygenase-2; DAGL, diacylglycerol lipase; eCB, endocannabinoid; FAAH, fatty acid amide hydrolase; GPR, G protein-coupled receptor; LOX, lipoxygenase; MAGL, monoacylglycerol lipase; NAAA, N-acylethanolamine hydrolyzing acid amidase; NAPE-PLD, N-acyl phosphatidylethanolamine-specific phospholipase D; PPAR, peroxisome proliferator-activated receptor; PTPN22, protein tyrosine phosphatase non-receptor type 22; THC, (-)-*trans*-Δ^9^-tetrahydrocannabinol; THCV, (-)-Δ^9^-tetrahydrocannabivarin; TRP, transient receptor potential ion channel.

In a wider sense, ECS comprises different metabotropic (e.g. CB_1_, CB_2_, GPR55, GPR119), ionotropic [members of the transient receptor potential (TRP) superfamily] as well as intranuclear cannabinoid-responsive receptors (e.g. PPAR), endogenous ligands [e.g. N-arachidonoylethanolamine (a.k.a. anandamide, AEA), 2-arachidonoylglycerol (2AG)] as well as the transporters [e.g. the putative endocannabinoid membrane transporter (EMT)] and enzymes involved in the synthesis [e.g. N-acyl phosphatidylethanolamine-specific phospholipase D (NAPE-PLD), diacylglycerol lipase (DAGL)α and β] and degradation [e.g. fatty acid amide hydrolase (FAAH)1 and 2, monoacylglycerol lipase (MAGL)] of the said ligands (**Figure 7**). Importantly, depending on the restrictiveness of the definition, several other cannabinoid-like molecules can also be classified as members of the ECS, or with a more inclusive term, the “endocannabinoidome” (193, 194). Obviously, not every ligand is capable of modulating the activity of every receptor listed on **Figure 7**. Instead, every ligand does have its own, unique, and concentration-dependent preferential target spectrum. Together with some other factors (e.g. biased signaling, receptor heteromerization (for details see references 185, 190) these differences explain the remarkable versatility of the cannabinoid signaling. Finally, one should not forget that, besides the endogenous ligands, several natural (classical and non-classical phytocannabinoids) and synthetic/semi-synthetic ligands can also modulate the activity of the system.

#### 5.4.2 Role of the complex (endo)cannabinoid signaling in the SG

**Figure 8:**
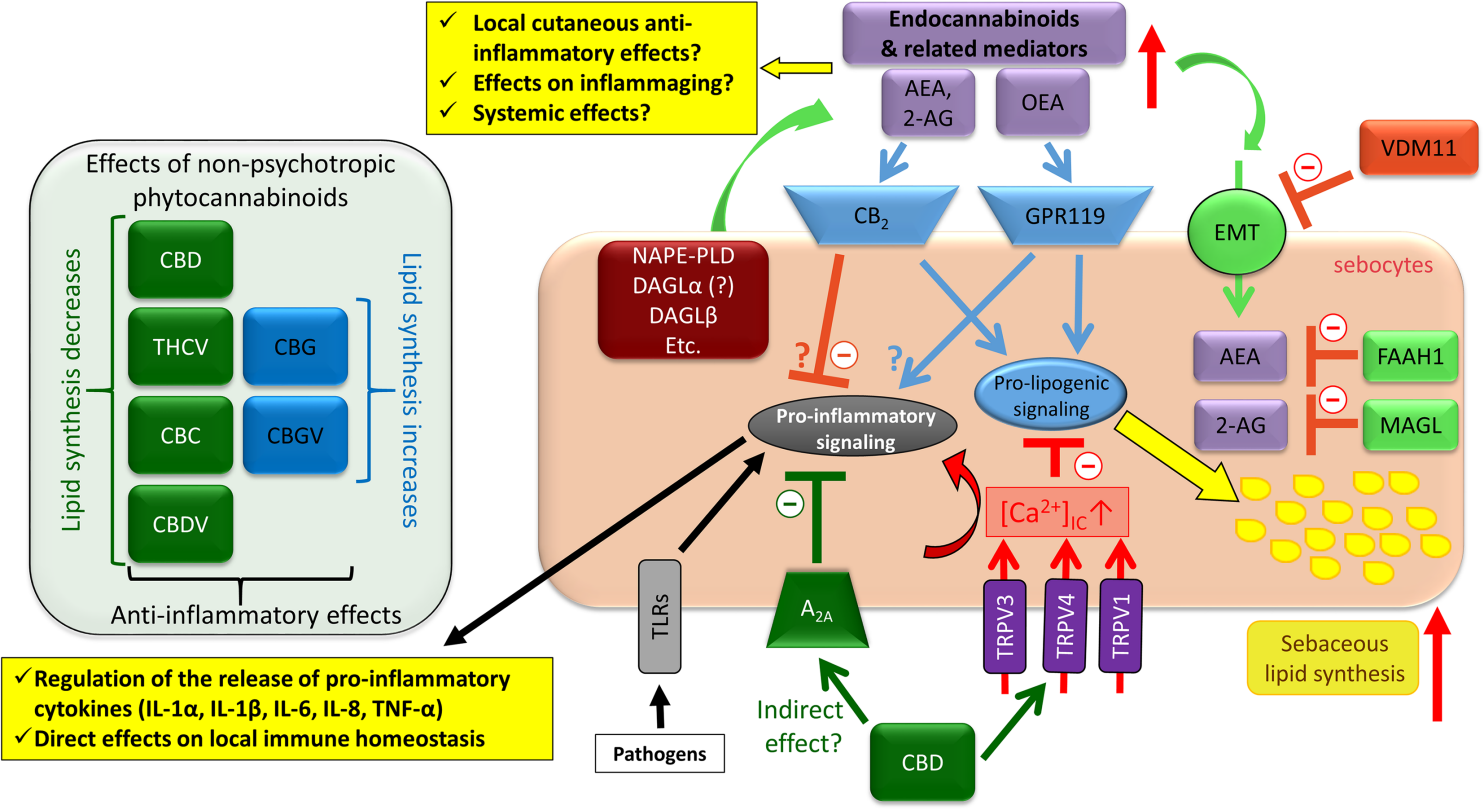
Overview of the effects of the complex cannabinoid signaling on human sebocytes. Sebocytes are able to metabolize endocannabinoids, and express multiple cannabinoid responsive receptors that contribute to the regulation of several aspects of the biology of human sebaceous glands. Importantly, *via* releasing various cytokines/chemokines as well as controlling the local anti-inflammatory endocannabinoid tone, sebocytes may be capable of shaping cutaneous immune responses. Note that besides CBD, THCV, CBC, and CBDV could also suppress excessive sebaceous lipogenesis, whereas CBG and CBGV induced a moderate increase in the lipid synthesis, and all tested phytocannabinoids were shown to exert anti-inflammatory effects. 2-AG, 2-arachidonoylglycerol; A_2A_, adenosine 2A receptor; AEA, anandamide; CBC, (-)-cannabichromene; CBD, (-)-cannabidiol; CBDV, (-)-cannabidivarin; CBG, (-)-cannabigerol; CBGV, (-)-cannabigerovarin; DAGL, diacylglycerol lipase; eCB, endocannabinoid; EMT, endocannabinoid membrane transporter; FAAH, fatty acid amide hydrolase; GPR, G protein-coupled receptor; IL, interleukin; MAGL, monoacylglycerol lipase; NAPE-PLD, N-acyl phosphatidylethanolamine-specific phospholipase D; OEA, oleoylethanolamide; PPAR, peroxisome proliferator-activated receptor; THCV, (-)-Δ^9^-tetrahydrocannabivarin; TLR, toll-like receptor; TNF, tumor necrosis factor; TRPV, transient receptor potential ion channels, vanilloid subfamily; VDM11, a pharmacological inhibitor of EMT.

The most important receptors, i.e. CB_1_ and CB_2_, were first shown to be expressed in human SG almost two decades ago (195). Indeed, CB_2_ is mostly expressed on the less differentiated cells of the basal layer, whereas CB_1_ is characteristic for the more differentiated cells (195, 196). Later, it was also shown that tonic auto- and paracrine activation of CB_2_ by the locally produced prototypical endocannabinoids (AEA and 2AG) plays a role in maintaining homeostatic sebaceous lipogenesis (196), while direct endocannabinoid treatment greatly enhanced lipid synthesis *via* activating CB_2_ receptor, the extracellular signal−regulated protein kinase (ERK)1/2 mitogen-activated protein kinase (MAPK) cascade, and PPARγ (194). Importantly, sebocytes were shown to express the most important enzymes involved in the synthesis (NAPE-PLD, DAGLα and β) and in the degradation (FAAH1, MAGL) of the endocannabinoids, and functional evidence argues that they also possess the putative EMT (197). Indeed, moderate elevation of the endocannabinoid-tone by the EMT-inhibitor VDM11 significantly increased sebaceous lipogenesis, and prevented the pro-inflammatory effect of the TLR4 activator lipopolysaccharide (LPS) (197). Although these data argue that, similar to many other cell types, endocannabinoid tone serves as a guardian of the inflammatory cytokine/chemokine production, the role of ECS appears to be more complex. Indeed, administration of oleoylethanolamide [OEA; an “ECS-related” molecule that was shown to be metabolized by the sebocytes (197)] promoted sebaceous lipogenesis *via* activating a recently deorphanized, novel endocannabinoid receptor (GPR119), as well as multiple signaling pathways [ERK1/2, c-Jun N-terminal kinase (JNK), CREB, and Akt (protein kinase B, PKB)] (198). Moreover, OEA also increased the production (IL1α, IL1β, IL6 and IL8) and release (IL6 and IL8) of several pro-inflammatory cytokines (198) (**Figure 8**).

As mentioned above, several members of the TRP ion channel superfamily (i.e. TRPA1, TRPM8, TRPV1, 2, 3, and 4) can be considered as ionotropic cannabinoid receptors (193, 199–201). Notably, TRPV1, 2, 3, and 4 are expressed on human sebocytes (202–204), whereas expression of TRPA1 and TRPM8 were found to be below detection limit (204). Importantly, unlike the previously mentioned members of the ECS, all tested TRPV were found to suppress sebaceous lipogenesis (202–204). Moreover, production (IL1α, IL1β, IL6, IL8 and TNFα) and release (IL1α, IL1β, IL6 and IL8) of several key pro-inflammatory cytokines were significantly elevated upon activation of TRPV3 (203). Although TRPV3 is a predominantly Ca^2+^-permeable ion channel that exerted its effects *via* Ca^2+^-coupled signaling events (203), Ca^2+^-influx does not necessarily evoke pro-inflammatory response in human sebocytes. Indeed, nicotinic acid (a member of the vitamin B_3_ complex) was found to suppress pro-acne agents-induced excessive sebaceous lipogenesis *via* activating hydroxycarboxylic acid receptor 2 (HCA_2_) (205). Although HCA_2_ is a metabotropic receptor, its activation led to Ca^2+^-influx that was indispensable for the development of its lipostatic effects (205). Interestingly, however, the said elevation of the intracellular Ca^2+^ concentration did not increase the production (IL1α, IL1β, IL6 and IL8) or release (IL6 and IL8) of key pro-inflammatory cytokines (205).

Importantly, complex cannabinoid signaling can also be targeted by exogenous agents, e.g. by phytocannabinoids. Indeed, (–)-cannabidiol (CBD; the best studied non-psychotropic phytocannabinoid) was shown to quantitatively and qualitatively normalize pro-acne agents-induced excessive sebaceous lipogenesis *via* activating TRPV4, and prevented the TLR4-activator LPS and the TLR2-activator lipoteichoic acid (LTA)-induced pro-inflammatory response through the most likely indirect activation of A_2A_ adenosine receptors (204) (**Figure 8**).

Moreover, besides CBD, several other phytocannabinoids, namely (–)-cannabigerol (CBG), (–)-cannabigerovarin (CBGV), (–)-cannabichromene (CBC), (–)-cannabidivarin (CBDV), and (–)-Δ^9^-tetrahydrocannabivarin (THCV) were also shown to exert anti-inflammatory effects on human sebocytes (206). Intriguingly, although all tested phytocannabinoids exerted anti-inflammatory effects, they exhibited a surprising functional heterogeneity in case of sebaceous lipogenesis. Indeed, CBG and CBGV behaved in an “endocannabinoid-like” manner, and slightly, but significantly elevated sebaceous lipogenesis (206). On the other hand, CBC and THCV decreased the spontaneous lipid production, whereas CBDV had no effect on it (206), but the arachidonic acid-induced, “acne-mimicking”, excessive sebaceous lipid production (204, 207) was efficiently suppressed by all three compounds (206), among which, THCV exerted “CBD-similar” anti-acne effects (206) (**Figure 8**).

#### 5.4.3 Modulation of the local eCB metabolism in the SG – A potent tool to shape local immune responses?

As summarized above, human sebocytes are not only targets of different cannabinoids, but they are also able to synthesize and degrade several endocannabinoids. Thus, one might hypothesize that they can act as regulators of the local cutaneous anti-inflammatory endocannabinoid tone. As mentioned above, cannabinoid signaling can regulate the activity of the immune system, and, in most [but importantly, not all; see (208)] cases, it exerts anti-inflammatory/immunosuppressive effects (186, 187, 191, 192, 209, 210). For example, local endocannabinoid tone was shown to limit mast cell activity in the skin and in other tissues as well *via* activating CB_1_ (211, 212). Likewise, inhibition of FAAH, i.e. the most important endocannabinoid degrading enzyme, led to anti-inflammatory effects on human epidermal keratinocytes, and alleviated atopic dermatitis-like inflammation of NC/Tnd mice (213). Thus, enhancing the local endocannabinoid tone by pharmacological inhibition of the degradation of endocannabinoids in the SG may provide a novel, previously unexplored tool to alleviate cutaneous inflammatory processes. Moreover, CB_1_^-/-^ animals (i.e. ones lacking homeostatic endocannabinoid signaling) were shown to suffer from an early onset aging-like cutaneous phenotype (214). Because homeostatic endocannabinoid signaling also regulates mitochondrial activity in various cell types (including epidermal keratinocytes, most likely *via* activating the mitochondrially expressed subset of CB_1_) (215), appropriate modulation of the endocannabinoid signaling may promise to be beneficial in preventing premature cellular senescence as well as inflammaging.

Thus, (endo)cannabinoid signaling of the human SG may contribute to the regulation of the innate (as well as adaptive) immune system in two major ways: i) *via* regulating cytokine/chemokine production of the sebocytes; and ii) *via* alterations of the endocannabinoid metabolism and hence local cutaneous endocannabinoid supply. Although several lines of evidence support this concept, certain key open questions still await being answered, namely: a) Can SG secrete endocannabinoids at biologically relevant concentrations to the blood stream to contribute to the regulation of the immune homeostasis at distant organs? B) Can SG secrete endocannabinoids locally at biologically relevant concentrations to regulate immune homeostasis of the pilosebaceous unit and the interfollicular epidermis? C) What is the role of the cytokines/chemokines released by the SG in various cutaneous inflammatory conditions?

## 6 Environmental influence on SG

Endocrine-disrupting chemicals, i.e. exogenous compounds that compete hormone activity through direct or indirect interaction with hormone receptors, exhibit immunotoxic and carcinogenic effects in the human skin (37, 216). The specific effects of environmental pollution on the SG through dioxins and polycyclic hydrocarbons, e.g. cigarette smoke benzo(a)pyrene, are exerted by binding AhR on human sebocytes (39) and inducing dysregulation of sebaceous lineage, which leads to abnormal differentiation of sebocyte progenitor cells towards keratinocytes (36), chronic inflammation with IL6 production and reduction of sebaceous lipogenesis (38, 39). The clinical picture is dominated by scarring (216, 217), which is documented as dioxin-induced acne or chloracne (218). Endemic occurrence of this characteristic clinical picture has occurred in the past after several accidents in chemical industry plants (216, 218). In addition, allergic skin diseases, disorders of skin pigmentation, skin cancer, and premature skin aging are disorders that may occur under the chronic influence of environmental pollutants.

## 7 The holocrine secretion is a cell-specific programmed cell death

Terminally differentiated sebocytes are characterized by lipid droplet accumulation, increased cell volume and nuclear degradation (219, 220), whereas nuclear degradation disputes the old mechanistic concept of cell bursting due to their increasing volume. Indeed, the mechanism of holocrine sebocyte secretion differs from apoptosis, necroptosis and cornification, being a multistep, cell-specific lysosomal DNase2-mediated mode of programmed cell death (40, 41). Histone 3 (221) and 4 (128) proteins are essential for the fate of sebocyte lineage and DNase2-mediated DNA degradation is required for the proteolysis of histone 3 in terminally differentiated sebocytes.

In addition, programmed sebocyte death is associated with autophagy, which provides undifferentiated sebocytes precious nutritive materials and makes sebocytes self-sufficient (42). Interestingly, enhanced sebocyte autophagy, regulates sebaceous lipogenesis, improves skin barrier function and reduces acne symptoms in acne-prone skin (222, 223).

## 8 SG inflammation and skin diseases

Whereas until a few years ago, seborrhea, acne and rosacea were the only diseases coupled to SG abnormalities, several medical conditions have been associated with the SG in the last years as a result of better understanding of the SG pathophysiology (4, 224–226). Examples of sebaceous gland-associated inflammatory diseases are presented in **Table 1**.

**Table 1:** Medical conditions associated with disorders of the sebaceous gland.

Acquired conditions and diseases
Acne vulgaris Seborrhea Seborrhea-acne-hirsutism-androgenetic alopecia (SAHA) syndrome Dioxin-induced acne Rosacea Psoriasis Atopic dermatitis Eosinophilic pustular folliculitis Hidradenitis suppurativa Androgenetic alopecia Cicatricial alopecia Lichen planopilaris Polycystic ovary (PCOS) Hyperandrogenism-insulin resistance-acanthosis nigricans (HAIRAN) syndrome Synovitis-acne-pustulosis-hyperostosis-osteitis (SAPHO) syndrome Benign (sebaceous adenoma, sebaceoma) and malignant (SG carcinoma) tumors
Congenital disorders*
Congenital adrenal hyperplasia (CAH) (OMIM 201910) Familial sebaceous gland hyperplasia (OMIM 601700) Nevus sebaceus of Jadassohn (OMIM 163200) Pyogenic arthritis-pyoderma gangrenosum-acne (PAPA) syndrome (OMIM 604416) Sebaceous nevus syndrome with hemimegalencephaly (OMIM 601359) Organoid nevus phakomatosis (OMIM 165630) Steatocystoma multiplex (OMIM 184500) Adenomatous polyposis of the colon (OMIM 175100) Muir-Torre syndrome (OMIM 158320) Ulerythema ophryogenes/keratosis pilaris (OMIM 604093) Apert syndrome (OMIM 101200)

*Online Mendelian Inheritance in Man (OMIM) is an online, comprehensive compendium of human genes and genetic phenotypes (http://www.ncbi.nlm.nih.gov/omim).

### 8.1 Inflammation-associated genes expressed by human sebocytes and acne

He et al. (227) performed a genome-wide association study of severe acne in a Chinese Han population comprising 1,056 patients and 1,056 controls and replicated 101 single nucleotide polymorphisms in an independent cohort of 1,860 patients and 3,660 controls. They identified two new susceptibility loci at 11p11.2 [*DDB2* (damage-specific DNA-binding protein 2)] and 1q24.2 [*SELL* (L-selectin)], which are involved in androgen metabolism, inflammation processes and scar formation in severe acne. In a further study, Wang et al. (228) found that variants in *SELL*, *MRPS36P2* (mitochondrial ribosomal protein S36 pseudogene 2), *TP63* (tumor protein P63), *DDB2*, *CACNA1H* (calcium voltage-gated channel subunit α1 H), *ADAM19* (A disintegrin and metalloproteinase 19), *GNAI1* (guanine nucleotide binding protein α-inhibiting 1), *CDH13* (cadherin 13) and *GABRG2* (γ-aminobutyric acid type A receptor subunit γ2) genes interact to increased the risk of severe inflammatory acne in the Chinese population. At last, Yang T et al. (229) have reported that the androgen-related genes *CYP21A2* and *CYP19A1* are associated with severe acne in patients from Southwest China.

On the other hand, Trivedi et al. (230) have conducted gene array expression profiling and compared lesional to non-lesional skin in acne patients and non-lesional skin from acne patients to skin from normal subjects. Within the acne patients, 211 genes were upregulated in lesional skin compared to non-lesional skin. The detection of matrix metalloproteinases, inflammatory cytokines and antimicrobial peptides was prominent, whereas *MMP1* (matrix metalloproteinase 1), *MMP3*, *IL8 DEFB4A* (hBD4) and *GZMB* (granzyme B) were the strongest differentially expressed genes (DEG).

Sebocytes produce inflammatory responses by secreting proinflammatory cytokines, chemokines and antimicrobial peptides following activation by pathogens and pathogen-associated molecular pattern recognition receptor ligands. Previous studies showed that important inflammatory molecules, such as IL6, IL8, TNFα, 5-lipoxygenase (5LOX) or cyclooxygenase 2 [COX2; a.k.a. prostaglandin-endoperoxide synthase (PTGS2)] are increased in lesional sebocytes of acne patients (97, 109).

*P. acnes* leads to inflammatory response in the pilosebaceous units in acne. Nagy et al. (108) compared the antimicrobial peptide and proinflammatory cytokine/chemokine expression at mRNA and protein levels of SZ95 sebocytes in response to coculture with isolates of *P. acnes* type IA and type IB as well as *Escherichia coli*-derived LPS. The authors found that *P. acnes* isolates or LPS induced the expression of the hBD2, IL8 and TNFα in SZ95 sebocytes, but had no effect on the TLR2, TLR4 and IL1α expression. They demonstrated that *P. acnes* may contribute to the recruitment of inflammatory infiltrate by modulating both IL8 and hBD2 expression.

Using quantitative reverse transcription (RT)-polymerase chain reaction (PCR) and enzyme-linked immunosorbent assay (ELISA), Huang et al. (231) evaluated the cytokine expression of SZ95 sebocytes stimulated with cell-free extracts of *P. acnes*. They found the secretion of IL8 and IL6 significantly upregulated. Furthermore, using the inhibitors of NFκB or p38 MAPK, neutralizing antibody or shRNA of *TLR2*, the authors demonstrated that the enhanced expression of IL8 in cell-free extract-stimulated sebocytes was activated by NFκB and p38 MAPK pathways through TLR2-dependent signaling in human SZ95 sebocytes.

Li et al. (91) observed that IL1β expression was upregulated in SG of acne lesions. After stimulating human sebocytes with *P. acnes*, the activation of caspase-1 and secretion of IL1β were enhanced significantly. Moreover, they demonstrated the expression of NLR family pyrin domain containing 3 (NLRP3) influence the production of IL1β in sebocytes. These results suggest that *P. acnes* activates the NLRP3 inflammasome in human sebocytes. *P. acnes*-induced IL1β activation in SG may have a role in combating skin infections and in acne pathogenesis.

Hyperglycemic diets can induce IGF1 production and IGF1 signaling contributes to the pathogenesis of acne. Kim et al. (232) measured changes in the expression of inflammatory biomarkers after the treatment of cultured sebocytes with IGF1 by PCR and ELISA. The expression levels of IL1β, IL6, IL8, TNFα and NFκB in cultured sebocytes were increased. The gene expression of these inflammatory biomarkers were decreased after NFκB inhibitor treatment. The gene expression levels of *IGF1R* (IGF1 receptor), *IGFBP2* (IGF-binding protein 2), *SREBP* and *PI3KCA* (phosphatidylinositol-4,5-bisphosphate 3-kinase, catalytic subunit α) increased in sebocytes treated with IGF1. Sebum production from cultured sebocytes was also increased. They suggest that IGF1 might participate in the pathogenesis of acne by increasing expression of inflammatory biomarkers and sebum production in sebocytes. Insulin and IGF1 stimulated the phosphoinositide-3-kinases (PI3K)/protein kinase B (Akt) cascade and increased forkhead box protein O1 (FoxO1) nuclear export. FoxO1 is a core element in the pathogenesis of acne which antagonizes the expression of SREBP1c, restraining the transactivation of androgen receptors to inhibits lipogenesis (233).

Androgens cause the proliferation of ductal keratinocytes in the follicular canal causing hypoxia. As a result, the aerotolerant anaerobe *P. acnes* grows well under hypoxia. Choi et al. (234) observed that hypoxia induces lipid accumulation in SZ95 sebocytes. *SREBP1* mRNA levels were reduced under hypoxic conditions. The expression of perilipin (PLIN)2, TNFα and IL6 was upregulated. TNFα induces the formation of lipid droplets in SZ95 human sebocytes (235). These results indicate that hypoxia regulates inflammatory mediators and induces lipid accumulation. RNA-Seq analyses of DEG in SZ95 sebocytes under hypoxia revealed 256 DEG, including several lipid droplet-associated ones. DEG between acne and non-acne skin are significantly enriched in hypoxia gene sets (234).

Sebum secretion is a major factor in the pathophysiology of acne. Treating the SZ95 sebocytes with palmitic acid, Choi et al. (145) found the intracellular lipid levels aswell as *IL6* and *IL8* mRNA levels and secretion were increased. Takata et al. observed that culturing SZ95 sebocytes in the presence of a blocking antibody against EGF receptor, increased the mRNA levels of *IL6*, *IL8* and *TNFα*, but have no influence on the level of *IL1α* (236). Starting from previous observation, Törőcsik et al. (20) cultivated SZ95 sebocytes with palmitic acid plus EGF, the cells lipid accumulation was decreased and IL6 level was increased. Compared the gene expression profile of SZ95 sebocytes cultured without EGF supplementation, the down-regulated 218 transcripts formed clusters related to steroid, retinoid and lipid metabolism, and to epidermal differentiation. Sebocytes co-treated with EGF plus palmitic acid enhanced the expression of *IL1* signaling related genes, such as *IL8* and *COX2*, when compared to sebocytes only treated with EGF or palmitic acid. They provided evidence that EGF and palmitic acid may contribute together to lipid accumulation and inflammation in sebocytes.

TNFα is an important pathophysiologic factor involved in the development of acne. Using RT-PCR and Western blot analyses, Choi et al. (145) found the levels of mRNA and protein expression of FA synthase (FAS) and SREBP1, as well as the level of phosphorylated p38 and c-Jun-N-terminal kinases (JNK) were significantly increased in TNFα-treated cells. After successful siRNA transfection-mediated silencing of Akt, SREBP1 and FAS expression as well as lipid synthesis were decreased in TNFα-treated SZ95 human sebocytes. SREBP are activated under stimulation. They bind FAS and this results in lipid formation (60). These results suggest that the PI3K/Akt pathway may be involved in TNFα-induced lipogenesis in SZ95 human sebocytes.

Sebocytes are not just a target of inflammation, but could actively contribute to skin homeostasis and the inflammatory environment. Choi et al. (145) reported oleic acid and linoleic acid promoted monocytes to differentiate into macrophages. Linoleic acid has anti-inflammatory effect and inhibits the secretion of IL1β, IL6 and TNFα. On the contrary, palmitic acid, stearic acid and oleic acid increase the secretion of IL1β. Oleic acid downregulated IL6 and TNFα secretion.

Palmitic acid is a valid stimulator of IL1β and TNFα cytokine production (19). Lovászi et al. (19) maintained macrophages in supernatant of SZ95 sebocytes co-cultured with *P. acnes* and found that linoleic acid also had an anti-inflammatory effect in *P. acnes*-activated macrophages, inhibiting the secretion of IL1β, IL6 and TNFα. Squalene, palmitic acid, stearic acid and oleic acid augmented the secretion of IL1β, even in the absence of *P. acnes*, whereas oleic acid had a selective effect of inducing IL1β but downregulating IL6 and TNFα secretion.

Mattii et al. (22) compared acne lesion and healthy skin-derived immunofluorescence stainings for CD4 and IL17 and found that CD4+ IL-17+ T cells surrounding the pilosebaceous unit were in close contact with sebocytes in acne lesions. They could also show that chemokines, such as IL8, CCL2, CCL5 and CXCL10, were abundant in cell-free supernatants from human SZ95 sebocytes. Moreover, the authors demonstrated that sebocytes had chemotactic effect on neutrophils, monocytes and T cells in an IL8-dependent manner and affected the differentiation of CD4+ CD45RA+ naïve T cells. Previous studies demonstrated that sebocytes secrete various cytokines, such as IL6 and IL1β, which are key cytokines for *de novo* differentiation of Th17 cells (237, 238). They concluded that sebocytes release cytokine and participate in inflammatory processes in the skin by recruiting and communicating with immune cells.

#### 8.1.1 SG modulation of the upper hair follicle and acne

Mature sebocytes burst and release sebum through the sebaceous duct into the hair canal, or infundibulum, which opens to the skin surface. Recent studies have shown that these interconnected domains of the upper hair follicle - the SG, sebaceous duct and infundibulum - share certain key molecular features, including expression of keratin 79 and the transcription factor GATA6 (35, 71, 239, 240). Mutant mice lacking either gene possess aberrant SG (35, 72), while Gata6-deficient mice additionally manifest expanded hair canals and sebaceous ducts (73). These phenotypes suggest tantalizing molecular connections between these follicular domains. These connections are further strengthened by the central role SG are thought to play in the pathogenesis of acne, which affects the follicular canal.

While the causes of acne are complex and likely multifactorial, the disease is characterized by several common features (4, 241, 242). Excessive sebum production, usually hormonally-driven, likely favors dysbiotic colonization of the hair follicle by *P. acnes*, causing inflammation that is further heightened by pro-inflammatory components found in sebum, such as triglycerides and lipoperoxides (243, 244). Alterations in sebum composition, including increased sebaceous lipids (free FA, squalene, squalene oxide) and reduced linoleic acid, may further drive hyperproliferation, hyperkeratinization and impaired differentiation in the infundibulum (106, 245–247), including reduced expression of keratin 79 and GATA6 (239, 240). Ultimately, these changes convert the upper hair follicle into a cyst-like comedone, the hallmark morphological feature of acne. In severe cases, the comedonal wall may rupture, releasing sebum into the dermis and further eliciting an inflammatory response (247).

These complex phenotypes suggest that SG activity and sebum composition profoundly influence upper hair follicle physiology, from proliferation to differentiation and desquamation. Sebum, which contains both pro- and anti-inflammatory factors, may modulate hair follicle keratinocytes directly, as well as indirectly by providing sustenance for microflora such as *P. acnes* (21, 248). Notably, isotretinoin, which is used to treat severe acne, causes a dramatic reduction in SG size and activity (249–251), further reinforcing the notion that SG play a central role in acne pathogenesis. Interestingly, and in contrast to acne, SG hypoplasia has been noted in other upper hair follicle pathologies, including chloracne, keratosis pilaris and hidradenitis suppurativa, which also form follicular cysts and occlusions (26, 252–254). Further studies are required to clarify whether SG disruption plays a functional role in these diseases.

### 8.2 Hair follicle regulation through the SG

In addition to promoting skin barrier function, SG have also been implicated in controlling proper hair follicle growth and homeostasis. Since SG are intimately and ubiquitously associated with hair follicles, this likely affords numerous opportunities for cross-regulation. Indeed, several disorders associated with hair follicle abnormalities, including scarring alopecia and acne, have been linked to SG dysfunction (4, 255, 256). While the instigating factors underlying these conditions have not been pinpointed, sebum components, including squalene and linoleic acid, can modulate keratinocyte cytokine production (119, 243) and may cause inflammation leading to follicular loss or deformation (256). Finally, since lipids function as critical signaling mediators in hair growth pathways such as Hedgehog and WNT (257–259), this may represent yet another way by which SG “talk” to hair follicles. Experimental and clinical evidence suggest that proper SG function is crucial for maintaining proper hair follicle physiology.

#### 8.2.1 SG modulation of hair shaft exit and scarring alopecia

Scarring or cicatricial alopecia is an inflammatory disorder where hair follicles are permanently lost and replaced with fibrous scar tissue (260). SG atrophy and destruction are thought to be early events in scarring alopecia (261), where inflammation is frequently concentrated near the upper follicle where SG typically reside (256, 262). Although it is unclear whether SG loss is causal for, or merely associated with, hair follicle destruction, lymphocytic attack of SG and follicular injury have also been observed in a mouse model of acute graft versus host disease (263), possibly suggesting common mechanisms in inflammation-mediated diseases.

Concordant with a causal role for SG loss leading to hair follicle destruction, the spontaneously arising asebia mutant mouse strain possesses hypoplastic SG and develops features resembling scarring alopecia, including patchy hair loss and inflammation (264, 265). Early studies in asebia mice, which lack the key sebaceous enzyme Scd1 (17, 23, 60, 61, 266), noted defects in hair shaft separation from its protective inner root sheath (264). With no way to exit the skin, entrapped growing hair shafts were thought to exert downward pressure, leading to rupture of the follicular base, dermal scarring, inflammation and eventual hair loss. Alopecia-like phenotypes have also been noted in other mutant strains with aberrant SG formation or sebum production, including mice lacking key sebaceous proteins such as FA 2-hydroxylase (Fa2h), diacylglycerol-O-acyltransferase 1 (Dgat1), cell death-inducing DFFA-like effector A (Cidea), ceramide synthase 4 (CerS4), gasdermin A3 (Gsdma3) and others (266–273). Frequent dilation and blockage of the follicular infundibulum by sebum plugs are commonly observed in these strains, likely causing hair shaft entrapment, as seen in asebia mice. These findings suggest that at least one way by which SG modulate follicular function is to release lytic factors, such as matrix metalloproteinases (249), which facilitate the unfettered egress of hair shafts from the skin. Interestingly, chemotherapy-induced alopecia has also been shown to be associated with abnormal sebaceous gland differentiation in experimental animals (274). These data confirm the concept of Stenn et al. (275), who have suggested the “sebogenic hypothesis” of pilosebaceous development. This hypothesis postulates that SG were, after nails, the first mammalian skin appendages to develop during the evolution and that the hair shaft, at least initially, solely served as a wick to draw sebum to the skin surface and disperse it there to enhance skin epidermal barrier.

### 8.3 SG atrophy and lichen planopilaris

SG atrophy and altered sebum composition are also observed in lichen planopilaris (LPP), a form of scarring alopecia where lipid metabolic changes appear to precede inflammation, suggesting a possible causal role for SG dysfunction in the said disease (256). LPP has been associated with downregulation of PPARγ, a key nuclear receptor-transcription factor that activates lipid metabolism gene expression (276). Mice lacking Pparγ fail to form SG (277), whereas animals with targeted deletion of Pparγ in hair follicle stem cells exhibit hair follicle destruction, perifollicular fibrosis and dermal inflammation, reminiscent of LPP (276). Although SG are dysmorphic in these animals during disease progression, it remains unclear whether hair follicle loss is directly attributable to abnormal SG in this model (276). Given that Pparγ serves an anti-inflammatory role in the skin (278), increased follicular inflammation caused by Pparγ deletion, rather than SG dysfunction, may very well underlie disease phenotypes. Thus, it is important to caution that while mice with aberrant SG may provide valuable insights into gland function, disease phenotypes observed in animal models may not necessarily recapitulate the root causes of diseases seen in patients.

However, there are still open questions and discrepancies in the existing data. Experimental studies in mice as well as clinical studies in patients have provided evidence that SG may control multiple aspects of follicular behavior, including hair shaft exit and upper hair follicle homeostasis. Nonetheless, the specific mechanisms by which abnormal SG and sebum contribute to pathology have not been fully elucidated, even for diseases such as acne. In animal models such as asebia mice, non-specific disruption of Scd1 complicates the ability to unequivocally link hair follicle defects to SG dysfunction. Indeed, recent studies utilizing a diphtheria toxin-mediated strategy to ablate sebocytes did not report major defects in hair growth or cycling (279). Plin2-deficient mice also possess hypoplastic SG while maintaining grossly normal skin (280). It is possible that the relative absence of hair phenotypes in these systems may be due to incomplete disruption of SG activity. Alternatively, hair phenotypes may not manifest until later in life, such as in Cidea-null mice, which develop hair loss only after 10 months of age (269). Other confounding questions include whether changes in SG size, which are frequently observed in skin disease, relate to alterations in SG activity and sebum composition. Perhaps most pressing, given the well-established differences in SG physiology and sebum composition between mouse and human (241, 281), further studies are needed to firmly establish a role for SG in hair follicle homeostasis, and to determine whether SG loss represents a primary or secondary event in such diseases.

### 8.4 Eosinophilic pustular folliculitis: an unexpected paradigm of SG-controlled immunological reaction

SG represent unique structure that usually annex to hair follicles. Lipids secreted by these glands constitute a part of the skin barrier function. SG produce antimicrobial peptides, cytokines, and chemokines that modulate skin immunity (282). SG play a pivotal role in pathological conditions. An example is IL17- producing T helper 17 (Th17) cells that accumulate around SG in lesion of acne vulgaris (22). In this pathological condition, *P. acnes*-activated sebocytes produce cytokines, including IL6, transforming growth factor (TGF)β and IL1β, to endow dermal dendritic cells with capability to prime Th17 cells (22).

**Figure 9:**
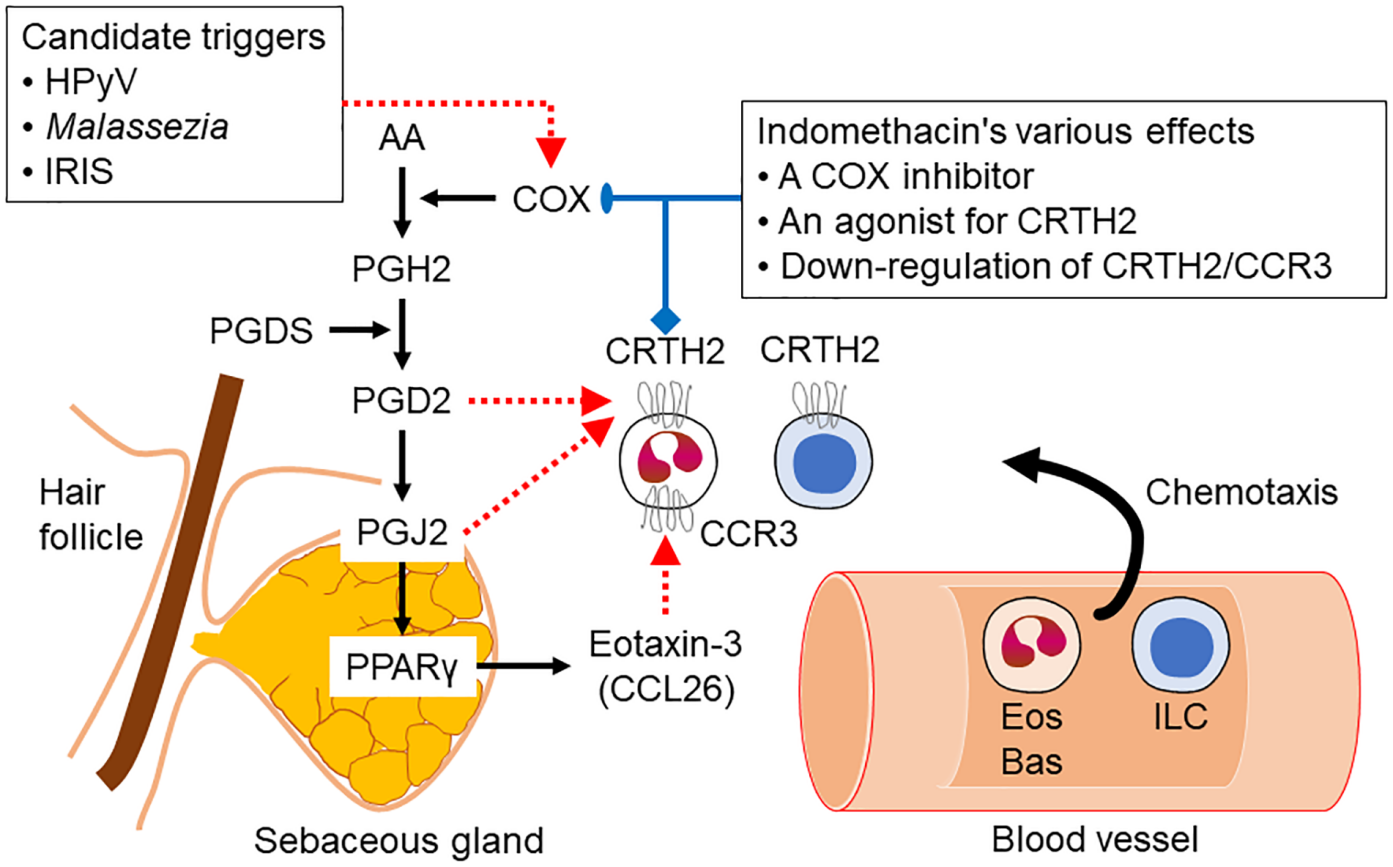
Eosinophilic pustular folliculitis: An unexpected paradigm of sebaceous gland-controlled immunological reaction. Infection with human polyoma virus or Malassezia as well as the immune reconstitution inflammatory syndrome stimulate the arachidonic acid inflammatory pathway in the sebaceous gland. Prostglandins, downstreem arachidonic acid metabolites, attract eosinophils and basophils to the perisebaceous gland dermis. Indomethacin might exhibit its therapeutic effect by inhibiting cyclooxygenase levels or blocking the chemoattractant receptor homologous molecule expressed on Th2 cells and the chemokine receptor 3. HPyV, human polyoma virus; IRIS, immune reconstitution inflammatory syndrome; AA, arachidonic acid; COX, cyclo-oxygenase; PG, prostaglandin; PGDS, PGD synthase; PPARγ, peroxisome proliferator-activated receptor γ; CRTH2, chemoattractant receptor homologous molecule expressed on Th2 cells; CCR3, C-C motif chemokine receptor 3; CCL26, C-C motif chemokine ligand 26; Eos, eosinophil; Bas, basophil; ILC, innate lymphoid cell.

Contrary to acne vulgaris in which Th17 cells and neutrophils play a role, eosinophilic pustular folliculitis (EPF) exhibits sterile eosinophilic pustules around the SG (283–285) (**Figure 9**). The massive accumulation of eosinophils in EPF is mediated by prostaglandin (PG)D2, which is presumed to be produced by PGD synthase of epithelial cells of pilosebaceous unit (286, 287). We have shown PGD2 and its immediate metabolite 15-deoxy-Δ-^12,14^-PGJ2 (15d-PGJ2) induced sebocytes to produce the chemoattractant CCL26, also known as eotaxin-3, *via* PPARγ (286). Pathologically high levels of eotaxin-3 produced by sebocytes are presumed to be the main driver for the accumulation of eosinophils.

#### 8.4.1 Concept of EPF pathogenesis

The concept of EPF was proposed by Ofuji as a sterile inflammation of the pilosebaceous unit accompanied by infiltration of numerous eosinophils (288). Clinically, it is an exudative inflammation characterized by centrifugal expansion of extensively pruritic papules and sterile pustules in an annular or plaque-forming pattern, mainly on the face and upper trunk (288, 289).

The efficacy of indomethacin, a non-steroidal anti-inflammatory drug, was reported in 1978, which suggested an association between EPF and PG (290, 291). On the other hand, activation of Th2-type immune response has also been suggested from cytokine patterns in serum of EPF patients (290, 292). Currently, PGD2/PGJ2 and chemoattractant receptor-homologous molecule expressed on Th2 cells (CRTH2), which is expressed by eosinophils, ILC2 and Th2 cells, are thought to play a central role (286, 293) (**Figure 9**). It is unclear why indomethacin is specifically effective in many cases of EPF. Indomethacin inhibits both COX enzymes, that synthesize prostanoids such as PGH2 from arachidonic acid. The fact that indomethacin binds to CRTH2, a PGD2 receptor, and suppresses the expression level of CRTH2 provides one explanation for its efficacy (293, 294). Interestingly, isotretinoin has been shown to be effective in EPF, which is an additional indication of SG involvement in EPF pathogenesis (295).

It is unknown why such Th2-type inflammation is triggered in patients with EPF. Suspected triggers include immune compromised state-mediated infection with *Malassezia* and immune reconstitution inflammatory syndrome (283, 285, 290). A study has added human polyomavirus 6 infection as an additional candidate for the culprit of EPF in Japanese (296). Worldwide studies are awaited to determine whether Asian/Japanese genotype human polyomavirus 6 is associated preferentially with the incidence and pathogenesis of EPF, which has an ethnic predilection for the East Asian population.

### 8.5 Atopic dermatitis and sebaceous lipids

Atopic dermatitis (AD) is associated with a defect in skin permeability barrier function, probably due to filaggrin mutations, leading to reduced protection against bacteria, irritants, allergens and environmental factors as well as modified microbiota (297). As a consequence an immune system response might be triggered resulting in skin inflammation. Epidermal lipids, such as ceramides, FA, triglycerides, and cholesterol, are integral components driving the formation and maintenance of the epidermal permeability barrier (298). Moreover, changes in skin surface lipids and a modified epidermal differentiation are important for the pathogenesis of AD (299).

Despite the knowledge that sebaceous lipids form 90% of the skin surface lipids in adolescent and adult individuals (1), it was only recently shown that the sebaceous gland density influences the stratum corneum lipidome (300). In addition to their contribution to the lipid barrier of the skin, sebaceous lipids also penetrate into the dermis (19–21), influence stromal cells and contribute to skin homeostasis by controlling the perifollicular ILC and commensal bacteria equilibrium (61, 90). Despite the dysfunctional synthesis of ceramides in keratinocytes (301) and the apparent skin xerosis in AD, the skin surface lipid concentration in patients with AD is marginally reduced but the proportion of sebaceous lipids is decreased and epidermal lipids (e.g. cholesterol) are increased (302, 303), a fact that might initiate cytokine release and perifollicular inflammation.

Interestingly, the AD-associated cytokines IL4 and IL13 were shown to stimulate the expression of 3β-HSD1, the key enzyme in sex steroid hormone metabolism, which is only expressed in SG in the skin (5, 157) and to induce lipid abnormalities in human sebocytes and keratinocytes *in vitro* (25). 3β-HSD1 expression is elevated in the skin of AD patients and can be restored by treatment with the IL4Rα monoclonal antibody dupilumab (25). Moreover, the antimicrobial proteins SPRR, whose synthesis and expression is abundant in the SG (27), were found to defend the skin permeability barrier by direct bacterial membrane disruption of the bacterial membrane (132). At last, sebaceous lipids, such as squalene (296) and propionic acid levels (303) are lower on the skin surface of AD patients compared with those of healthy individuals. In mice lacking sebum production with or without MC903-induced AD-like dermatitis, propionic acid attenuated skin inflammation (61, 303). Propionic acid reduced skin inflammation by inhibiting IL33 production in keratinocytes, an effect that was mediated through inhibition of histone deacetylase (HDAC) and regulation of the AhR signaling pathway (303). A proof-of-concept clinical study further demonstrated the beneficial therapeutic effects of topical propionic acid application in AD patients (303).

## 9 Conclusion

Half a century after the aphorism of the American master of dermatology, Prof. Albert M. Kligman (304) “The SG is … a living fossil with a past but not future”, the SG has advanced to “the brain of the skin” (15). A considerable amount of research has evidenced that this tiny, only 2% of the adult body mass counting but very active organ, with a particularly high energy requirement, is responsible or contributes markedly to numerous essential homeostatic functions for skin health and integrity (1–23). In addition to its important role in the developmental biology of the pilosebaceous unit (61, 274, 275), the SG and its major product, the sebaceous lipids, perform major contributions in skin physiology, endocrinology and immunology with the latter been a new fascinating research field (2, 4). The SG is involved in skin detoxification, cancer protection, regulation of bacterial colonization, neuroendocrinology and the innate immunity of the skin, properties which are summarized under the term “sebaceous immunobiology” (21). In addition to its role in seborrhea and acne, the SG seems to contribute in the development of several common inflammatory skin diseases, including atopic dermatitis, and possible rosacea, psoriasis and hidradenitis suppurativa (**Table 1**), and can, therefore, be considered as an important target for future therapeutic developments.

## Author contributions

CZ wrote the chapters 1 Introduction, 3.1 Bacterial antigens, pattern recognition receptors and SG, 5.3 Neuropeptides and SG immunity, 6 Environmental influence on SG, 7 The holocrine secretion is a cell-specific programmed cell death, 8 SG inflammation and skin diseases (Introduction), 8.1 Inflammation-associated genes expressed by human sebocytes and acne (partial contribution), 8.5 Atopic dermatitis and sebaceous lipids and 9 Conclusion; TC wrote the chapter 3.2 Bacterial biofilm and SG inflammatory response; LH wrote the chapter 8.1 Inflammation-associated genes expressed by human sebocytes and acne; KK and TN wrote the chapter 8.4 Eosinophilic pustular folliculitis: An unexpected paradigm of SG-controlled immunological reaction; TK wrote the chapter 4.2 Cross-regulation of SG and immune cells; CN wrote the chapters 2.1 SG stem and progenitor cells in development, homeostasis and pathologies, 2.2 SG formation and hair follicle morphology are closely linked, 2.4 Stem cells in SG homeostasis and 2.6 Future aspects of SG developmental biology; CN and SQ wrote the chapter 2.5 SG stem cells and cancer; AO wrote the chapter 5.4 Cannabinoids and inflammation in sebaceous glands – A “high”-way to heal?; MP wrote the chapter 5.2 PPAR and SG function; HS wrote the chapter 5.1 *In situ* production and action of sex steroids in human SG physiology and pathology; SQ wrote the chapter 2.3 Dermal progenitor cell markers in the stroma of SG; MS and DT wrote the chapters 4.1 Innate defense mechanisms of the SG and 4.3 Integrating sebaceous lipids into sebaceous immunobiology; SW wrote the chapters 8.1.1 SG modulation of the upper hair follicle and acne, 8.2 Hair follicle regulation through the SG and 8.3 SG atrophy and lichen planopilaris. All authors contributed to the article and approved the submitted version.

## Funding

TK’s work is supported by KAKENHI (grant numbers 20H03705 and 20K21534) from the Japan Society for the Promotion of Science, AMED (grant number JP21gm6510005), LEO Foundation Grant and Takeda Science Foundation. CN is grateful for financial support by the German Research Foundation (SFB829, Project-ID 73111208) and the Center for Molecular Medicine Cologne (CMMC). Her research is supported by the Koeln Fortune Program/Faculty of Medicine, University of Cologne. AO’s and DT’s work is supported by the National Research, Development and Innovation Office [grant numbers: 134235 (AO) and 132296 (DT)]; AO is recipient of the János Bolyai Research Scholarship of the Hungarian Academy of Sciences (ID: BO/00660/21/5), and was supported by the New National Excellence Program of the Ministry for Culture and Innovation from the source of the National Research, Development and Innovation Fund (“Bolyai+ Scholarship”) under the grant ID ÚNKP-22-5-DE-427. SYW is supported by the National Institutes of Health, USA (R01AR065409 and R01AR080654) and by the American Cancer Society (TLC-21-161-01-TLC).

## Conflict of interest

CZ reports thematically relevant, but current work independent, consultancy/advisory boards honoraria from AccureAcne, Almirall, Galderma, General Topics, GSK/Stiefel, L´ORÉAL, Luvos, NAOS-BIODERMA, Pierre Fabre and PPM; he is chair of the ARHS Task Force group of the EADV and Editor of the EADV News. AO provides consultancy services to Monasterium Laboratory Skin & Hair Research Solutions GmbH. MP received a research grant from PPM and provided consultancy to Incyte and Pfizer.

The remaining authors declare that the current work was conducted in the absence of any commercial or financial relationships that could be construed as a potential conflict of interest.

## Publisher’s note

All claims expressed in this article are solely those of the authors and do not necessarily represent those of their affiliated organizations, or those of the publisher, the editors and the reviewers. Any product that may be evaluated in this article, or claim that may be made by its manufacturer, is not guaranteed or endorsed by the publisher.
